# Incidence of Opportunistic Infections and the Impact of Antiretroviral Therapy Among HIV-Infected Adults in Low- and Middle-Income Countries: A Systematic Review and Meta-analysis

**DOI:** 10.1093/cid/ciw125

**Published:** 2016-03-06

**Authors:** Andrea Low, Georgios Gavriilidis, Natasha Larke, Marie-Renee B-Lajoie, Olivier Drouin, John Stover, Lulu Muhe, Philippa Easterbrook

**Affiliations:** 1 Clinical Research Department, Faculty of Infectious and Tropical Diseases, London School of Hygiene and Tropical Medicine, United Kingdom; 2 International Center for AIDS Care and Treatment Programs, Columbia University, New York, New York; 3 HIV Department, World Health Organization, Geneva, Switzerland; 4 Medical Research Council Tropical Epidemiology Group, London School of Hygiene and Tropical Medicine, United Kingdom; 5 Department of Family Medicine; 6 Department of Pediatrics, McGill University, Montreal, Canada; 7 Department of Centerfor Modeling and Analysis, Avenir Health, Glastonbury, Connecticut; 8 Department of Maternal, Child, and Adolescent Health, World Health Organization, Geneva, Switzerland

**Keywords:** HIV-1, antiretroviral therapy, opportunistic infections, tuberculosis, low- and middle-income countries

## Abstract

***Background.*** To understand regional burdens and inform delivery of health services, we conducted a systematic review and meta-analysis to evaluate the effect of antiretroviral therapy (ART) on incidence of key opportunistic infections (OIs) in human immunodeficiency virus (HIV)–infected adults in low- and middle-income countries (LMICs).

***Methods.*** Eligible studies describing the cumulative incidence of OIs and proportion on ART from 1990 to November 2013 were identified using multiple databases. Summary incident risks for the ART-naive period, and during and after the first year of ART, were calculated using random-effects meta-analyses. Summary estimates from ART subgroups were compared using meta-regression. The number of OI cases and associated costs averted if ART was initiated at a CD4 count ≥200 cells/µL were estimated using Joint United Nations Programme on HIV/AIDS (UNAIDS) country estimates and global average OI treatment cost per case.

***Results.*** We identified 7965 citations, and included 126 studies describing 491 608 HIV-infected persons. In ART-naive patients, summary risk was highest (>5%) for oral candidiasis, tuberculosis, herpes zoster, and bacterial pneumonia. The reduction in incidence was greatest for all OIs during the first 12 months of ART (range, 57%–91%) except for tuberculosis, and was largest for oral candidiasis, *Pneumocystis* pneumonia, and toxoplasmosis. Earlier ART was estimated to have averted 857 828 cases in 2013 (95% confidence interval [CI], 828 032–874 853), with cost savings of $46.7 million (95% CI, $43.8–$49.4 million).

***Conclusions.*** There was a major reduction in risk for most OIs with ART use in LMICs, with the greatest effect seen in the first year of treatment. ART has resulted in substantial cost savings from OIs averted.

## Abstract (precis)

This meta-analysis provides strong evidence that antiretroviral therapy (ART) has led to a major reduction in the incidence of key opportunistic infections (OIs) in low- and middle-income countries. ART was estimated to have averted about a million cases of OIs in 2013.

**(See the Major Article by B-Jajoie et al on pages 1586–94.)**

The impact of the introduction of highly active antiretroviral therapy (ART) on the incidence and mortality of human immunodeficiency virus (HIV)–associated opportunistic infections (OIs) has been well documented in high-income countries (HICs). This improved survival has been associated with a progressive shift in the pattern of comorbidities, with an increasing contribution of chronic liver disease due to hepatitis C and B, cardiovascular disease, and non-AIDS malignancies [1–4].

In low- and middle-income countries (LMICs), the global rollout of ART has led to >15 million patients on ART, and a decline in HIV-related deaths by 40% since 2004 [5, 6]. OIs remain the major driver of HIV-associated morbidity and mortality, accounting for the substantially higher mortality observed in LMICs [7], but the ART impact on specific OIs has not been well documented, mainly because there is no routine country-level monitoring for OIs.

Reliable data on the relative burden of different OIs before and after ART initiation in different geographic regions is critical for planning of health services, including procurement of relevant drugs and diagnostics. The few previous reviews on OI incidence either preceded the global scale-up of ART or addressed only specific OIs or regions [8, 9]. We have undertaken a comprehensive systematic review and meta-analysis to estimate the incidence of key OIs in HIV-infected adults before and after the introduction of ART in LMICs across 3 regions (sub-Saharan Africa, Asia, and Latin America and the Caribbean), and to evaluate the magnitude of effect of ART on the incidence of these OIs during and after the first year of treatment.

## METHODS

### Search Strategy and Selection Criteria

A systematic review of the literature was performed using Medline, Embase, Cumulative Index to Nursing and Allied Health Literature, Latin American and Caribbean Health Sciences Literature, Global Health, CAB Abstracts, Web of Science, and the Cochrane Library of Systematic Reviews from January 1990 to 21 November 2013, and conference proceedings (Conferences on Retroviruses and Opportunistic Infections, Google, and Web of Science) from 1997 to 2010, published in English, Spanish, French, and Portuguese. The gray literature was searched using Google Scholar and key sources such as the World Health Organization (WHO) website. Eligible studies documented the incidence of 16 OIs and coinfections among HIV type 1–infected adults (aged ≥18 years) in LMICs, as defined by the 2009 World Bank classification [10]. Our search was conducted according to Meta-analysis of Observational Studies in Epidemiology and Preferred Reporting Items for Systematic Reviews and Meta-analyses guidelines and is reported in Supplementary Appendix 1 [11, 12]. Key OIs included cryptococcal meningitis, *Pneumocystis* pneumonia (PCP), oral and esophageal candidiasis, cerebral toxoplasmosis, cryptosporidial diarrhea, genital ulcer disease (GUD) attributed to herpes simplex virus, herpes zoster, Kaposi sarcoma, cytomegalovirus retinitis, bacterial infections (pneumonia, enteritis, and bacteremia), and *Mycobacterium tuberculosis* (hereafter “tuberculosis”). Tuberculosis was classified as unspecified forms where data was only provided on the incidence of “tuberculosis,” and pulmonary tuberculosis (PTB) and extrapulmonary tuberculosis (EPTB), where these forms were specified. Criteria for OI diagnoses are described in Supplementary Appendix 2.

Eligible studies of incidence included prospective and retrospective observational cohort studies, and randomized controlled trials (RCTs). Where the RCT involved evaluation of a treatment for the OI under study, only data from the placebo arm were used.

All titles and abstracts were screened by independent reviewers (A. L. and G. G.) to identify potentially relevant articles. The full articles were eligible if they provided data from which a cumulative incident risk for individual OIs could be calculated. We excluded studies that included pregnant women or children, where the majority of patients had HIV type 2, were based on preselected populations such as those with a diagnosis of meningitis or pneumonia, or those with no reliable denominator. Studies where only repeated measures were reported and the incident risk of the first event could not be calculated were excluded. Where there were multiple reports from 1 cohort, we selected the most recent report with the longest follow-up. Data extracted for each study included study design, duration of follow-up, ART status, use of cotrimoxazole (CTX) prophylaxis, baseline CD4 counts, and diagnostic methods (Supplementary Appendix 2).

### Study Definitions

ART status was categorized as either “ART naive” (including studies with <10% of patients on ART, or when conducted prior to the availability of ART), or “ART exposed” (including studies with ≥80% of patients on ART). We excluded studies where the proportion on ART was ≥10% and <80%, or where data on ART use were not provided. To determine the relative impact of ART at lower CD4 counts when there is an increased risk of IRIS, the ART-exposed category was further stratified according to duration of time on ART: during the first year on ART, after the first year of ART, and during an unspecified time on ART where it spanned both periods or was not stated. Multiple estimates were extracted from articles if results were stratified according to ART status. Where incidence was provided according to different CD4 count strata, an overall estimate of incidence was presented based on the mean or median CD4 count of the population.

### Statistical Analyses

Since few studies reported incidence rates using person-years at risk, we calculated a cumulative incident risk of developing a specific OI (termed “risk” hereafter) for each study, defined as the cumulative number of new cases during follow-up divided by the number of persons at risk, and presented this as a percentage. Ninety-five percent confidence intervals (95% CIs) were extracted from the article, or calculated from raw data if not reported.

To estimate summary risks for each OI across studies, the variances of raw percentages were stabilized using Tukey–Freeman arcsine square root transformation [13]. Summary risks were generated using random-effects meta-analysis to adjust for high between-study variability. Between-study heterogeneity was evaluated using *I*^2^ and the *P* value for heterogeneity (Cochran Q statistic) [14]. Meta-analyses were performed using R software version 2.13.1. For OIs with data from ≥15 studies, we performed meta-regression analyses to calculate an adjusted odds ratio (aOR) and estimate the effect of each ART category on risk. The summary risks were transformed using empirical logistic transformation and univariable meta-regression was performed. ART categories with <3 studies were not included in the meta-regression.

In addition, we used meta-regression to explore potential sources of heterogeneity, including region, duration of time on ART, use of CTX prophylaxis, baseline CD4 count, method of OI diagnosis, and duration of follow-up. Univariable and multivariable meta-regression analyses were performed: time on ART, median follow-up time, and region were included a priori in multivariable models, and any variables from the univariable analysis with a *P* value *P* ≤ .20. Those variables that remained significant with a *P* ≤ .05 were retained in the final model. Meta-regression was performed using Stata software version 13. Meta-regression was also used to compare regional incidence of OIs across ART categories, for OIs for which there was at least 1 study per region, in the same manner as for the ART analysis.

### OI Cases Averted by the Use of ART

The estimated number of HIV-infected adults with CD4 counts <200 cells/µL for 156 LMICs were obtained from 2013 Joint United Nations Programme on HIV/AIDS (UNAIDS) country estimates [15–17]. The number of OI cases averted through use of ART for the year 2013 was calculated by applying the difference in estimates of OI risk in ART-naive participants and in those during their first year of ART to the estimated population with a CD4 count ≤200 cells/µL, for each region. The annual savings per OI averted were calculated for OIs where there was some evidence from the meta-analysis of an effect of ART (*P* < .20), and where there was data available for an average treatment cost per case [18], by multiplying the number of OI cases averted by the treatment cost per case. Uncertainty ranges were estimated from 1000 Monte Carlo draws of the difference in estimates of risk, assuming a normal distribution.

## RESULTS

**Figure 1.**
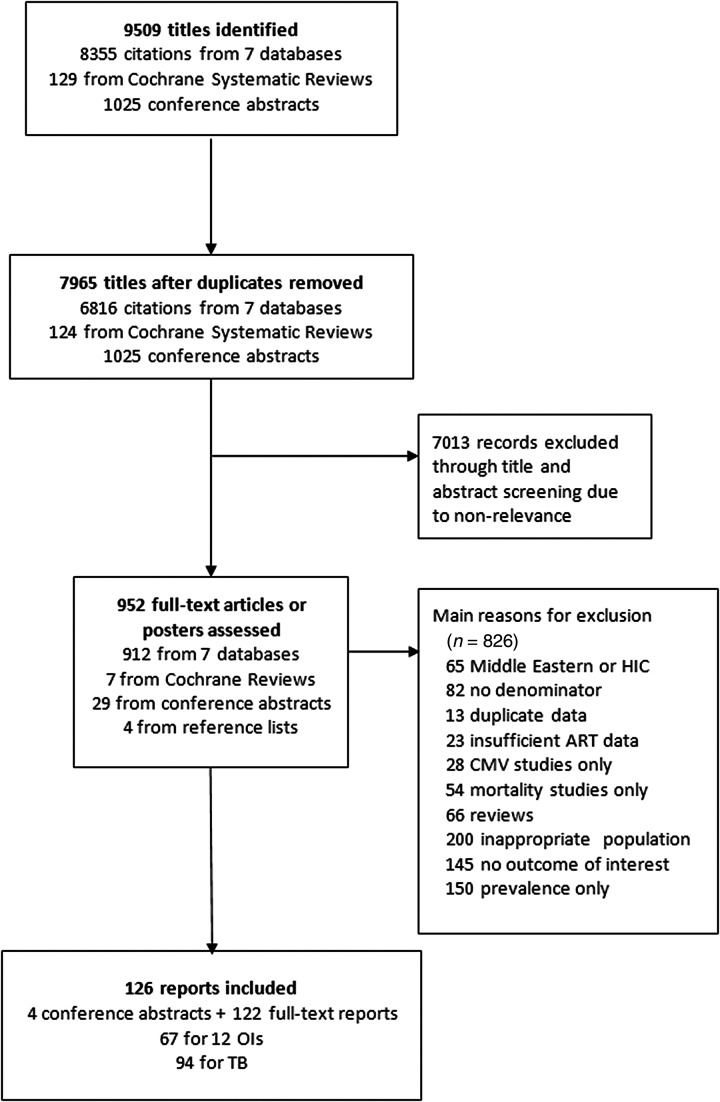
Flowchart of study selection. Abbreviations: ART, antiretroviral therapy; CMV, cytomegalovirus; HIC, high-income country; OI, opportunistic infection; TB, tuberculosis.

**Figure 2.**
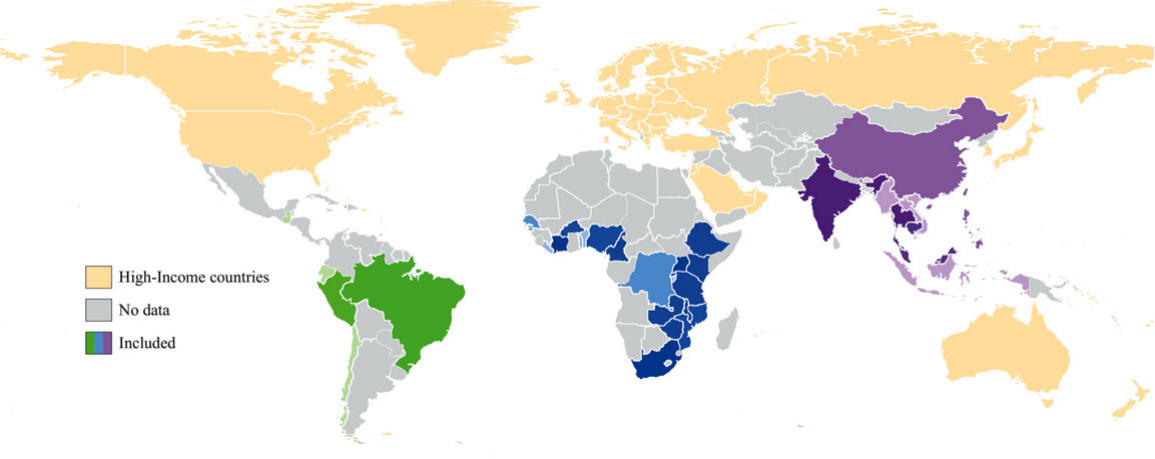
Low- and middle-income countries reporting the incidence of key opportunistic infections and antiretroviral therapy status. Thirty-eight countries provided included data. Green represents Latin America and the Caribbean, blue represents sub-Saharan Africa, and purple represents Asia. Color darkness reflects the number of included studies, with darker shading representing more studies. High-income countries were not eligible for inclusion.

The search strategy identified 6816 journal citations and 1025 conference abstracts; 952 full text reports were screened and 126 studies met the inclusion criteria (122 full-text articles and 4 conference abstracts; Figure 1). There were 67 studies describing the incidence of 12 OIs, and 94 studies for the different types of tuberculosis; 28 studies describing cytomegalovirus were excluded due to widely varying case definitions. Table 1 summarizes characteristics of included studies, which described data from 89 cohorts or trials in 38 countries; 85 (67%) studies were from sub-Saharan Africa (Figure 2). A total of 491 608 participants were included in our analysis: 432 469 patients in sub-Saharan Africa, 42 243 in Asia, and 16 896 in Latin America (Table 1). Study size ranged from 54 to 175 212 participants (median, 765 [interquartile range {IQR}, 248–1646]). Length of follow-up was available for 67 studies (53%), and ranged from 3 to 78 months (median, 24 months [IQR, 12–35 months]). A CD4 count <200 cells/µL, or the presence of a WHO stage III or IV disease, were the criteria for ART initiation in most studies. Laboratory or radiological confirmation was available in >75% of studies for diagnosis of *Cryptosporidium* diarrhea, tuberculosis, and bacterial infections, and in <50% of studies for candidiasis, herpes zoster, GUD, Kaposi sarcoma, and cerebral toxoplasmosis.

**Table 1.** Characteristics of Included Studies Describing Opportunistic Infections, by Region

Characteristic	Study Design	No. of Study Patients (Range)	Baseline CD4 Count, Range, Cells/µL	Laboratory/Radiological Confirmation^a^, no./No. (%)
Cryptococcal meningitis				
Sub-Saharan Africa	4 RCT, 12 cohorts	186 899 (60–175 212)	16–461	9/16 (56)
Asia	1 RCT, 8 cohorts	16 655 (54–10 904)	8–227	5/9 (56)
LAC	3 cohorts	1740 (318–1057)	64–208	2/3 (66)
*Pneumocystis* pneumonia				
Sub-Saharan Africa	1 RCT, 7 cohorts	183 619 (270–175 212)	128–499	5/8 (63)
Asia	9 cohorts	10 902 (54–5040)	26–130	7/9 (78)
LAC	6 cohorts	2420 (123–1057)	<200–492	3/6 (50)
Oral candidiasis				
Sub-Saharan Africa	2 RCT, 8 cohorts	20 531 (110–8409)	200–527	1/10 (10)
Asia	1 RCT, 6 cohorts	4152 (66–1982)	73–227	1/7 (14)
LAC	2 cohorts	1217 (160–1057)	<200–477	0/2
Esophageal candidiasis				
Sub-Saharan Africa	3 RCT, 5 cohorts	8627 (248–2446)	<100–322	1/8 (13)
Asia	1 RCT, 3 cohorts	2005 (54–1246)	43–73	0/4
LAC	2 cohorts	478 (160–318)	477	0/2
Herpes zoster				
Sub-Saharan Africa	1 RCT, 7 cohorts	4305 (101–1620)	135–461	0/8
Asia	4 cohorts	2457 (108–1253)	115–227	0/4
LAC	2 cohorts	1217 (160–1957)	<200–477	0/2
Genital herpes simplex or ulcer disease				
Sub-Saharan Africa	4 RCT, 7 cohorts	4916 (60–1215)	128–500	2/11 (18)
Asia	5 cohorts	5098 (54–1503)	43–129	0/5
LAC	1 cohort	318	208	0/1
Kaposi sarcoma				
Sub-Saharan Africa	2 RCT, 15 cohorts	275 298 (60–175 212)	118–461	3/17 (18)
Asia	1 cohort	76	NA	0/1
LAC	4 cohorts	2137 (145–1057)	183–492	0/4
Cerebral toxoplasmosis				
Sub-Saharan Africa	1 RCT, 9 cohorts	6914 (124–1620)	128–461	5/10 (50)
Asia	5 cohorts	3087 (54–1246)	43	2/5 (40)
LAC	4 cohorts	1680 (145–1057)	<200–492	1/4 (25)
*Cryptosporidium* diarrhea				
Sub-Saharan Africa	1 RCT, 5 cohorts	4413 (248–1215)	128–252	5/6 (83)
Asia	4 cohorts	1484 (54–1276)	43	3/4 (75)
LAC	1 cohort	160	477	1/1 (100)
*Mycobacterium tuberculosis* (unspecified)				
Sub-Saharan Africa	10 RCT, 42 cohorts	131 490 (60–14 422)	65–499	44/52 (85)
Asia	15 cohorts	24 496 (54–5099)	23–783	10/15 (67)
LAC	1 RCT, 9 cohorts	15 425 (60–8128)	105–492	7/10 (70)
Pulmonary tuberculosis				
Sub-Saharan Africa	1 RCT, 23 cohorts	62 884 (53–5980)	50–461	19/24 (79)
Asia	1 RCT, 8 cohorts	6972 (76–2984)	26–350	7/9 (78)
Extrapulmonary tuberculosis				
Sub-Saharan Africa	1 RCT, 19 cohorts	58 926 (86–5980)	50–461	17/20 (85)
Asia	8 cohorts	6772 (76–2984)	26–350	6/8 (75)
LAC	1 cohort	617	NA	1/1 (100)
Bacterial pneumonia				
Sub-Saharan Africa	4 RCT, 9 cohorts	6277 (53–1792)	200–461	13/13 (100)
Asia	1 cohort	134	NA	1/1 (100)
Isolated bacteremia				
Sub-Saharan Africa	2 RCT, 6 cohorts	2312 (101–792)	135–461	8/8 (100)
Asia	1 cohort	108	<200	1/1 (100)
Bacterial enteritis				
Sub-Saharan Africa	2 RCT, 7 cohorts	5603 (101–1792)	135–461	9/9 (100)

Clinical response to empiric treatment without imaging or laboratory confirmation or unspecified means of diagnosis is considered non–laboratory confirmed.

Abbreviations: LAC, Latin America and the Caribbean; NA, data not available; RCT, randomized controlled trial.

^a^ Laboratory confirmation for cryptococcal meningitis: cerebrospinal fluid cryptococcal antigen or India ink; for *Pneumocystis* pneumonia: chest radiograph and sputum cytology or clinical response; for oral candidiasis: fungal culture; for esophageal candidiasis: endoscopy and fungal culture; for herpes zoster or genital herpes simplex: Tzanck smear or polymerase chain reaction assay; for Kaposi sarcoma: biopsy and histology; for cerebral toxoplasmosis: computed tomographic brain scan, serology, and response to treatment; for *Cryptosporidium* diarrhea: modified Ziehl–Neelsen stool stain; for all forms of tuberculosis: acid-fast bacilli stain and/or mycobacterial culture; for all bacterial diseases: blood, sputum or stool cultures; for bacterial pneumonia: chest radiograph.

### Incidence of OIs in ART-Naive Participants

**Figure 3.**
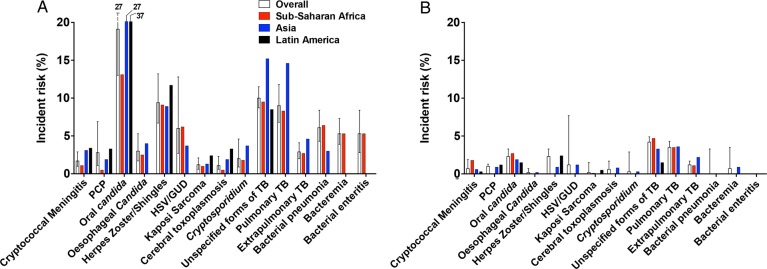
Summary incident risk of each opportunistic infection by region and overall for antiretroviral therapy (ART)–naive patients (*A*), and during the first year of ART (*B*). Abbreviations: GUD, genital ulcer disease; HSV, herpes simplex virus; PCP, *Pneumocystis* pneumonia; TB, tuberculosis.

The most common OIs in ART-naive participants were oral candidiasis (19.1% [95% CI, 13.0%–27.3%]), unspecified tuberculosis (10.0% [95% CI, 8.7%–11.5%]), herpes zoster (9.4% [95% CI, 6.7%–13.2%]), PTB (9.0% [95% CI, 6.8%–11.8%]), bacterial pneumonia (6.1% [95% CI, 4.3%–8.4%]), and GUD (6.0% [95% CI, 2.7%–12.8%]) (Table 2 and Figure 3A). There were some differences in incidence across the regions based on a comparison of the 11 OIs and coinfections for which there was at least 1 study per region (see Supplementary Appendix 3 and Supplementary Figure 1*A–O* for region-specific incidence and 95% CI). There were no data available in ART-naive participants in Latin America for esophageal candidiasis, GUD, *Cryptosporidium*, PTB, or bacterial infections, or in Asia for bacteremia or enteritis. Overall, there was a slightly lower reported risk of all OIs except GUD and bacterial pneumonia in sub-Saharan Africa; unspecified tuberculosis was most commonly diagnosed in Asia (15.2% [95% CI, 7.8%–27.6%]), and PCP (8.4% [95% CI, .5%–24.5%]), toxoplasmosis (3.3% [95% CI, 2.4%–4.4%]), and Kaposi sarcoma (2.4% [95% CI, .7%–8.0%]) in Latin America.

**Table 2.** Estimated Summary Incident Risks for Opportunistic Infections, by Antiretroviral Status and Duration of Antiretroviral Therapy Use

Opportunistic Infection	Summary of Incident Risk, % (95% CI) (No. of Studies)				Adjusted Odds Ratio^a,b^ (95% CI)			*P* Value^b^
	ART-Naive	<1 y ART	≥1 y ART	Unspecified Time on ART	<1 y ART	≥1 y ART	Unspecified Time on ART	
*Cryptococcal* meningitis	1.7 (1.0–2.9) (16)	0.7 (.3–1.9) (4)	6.9 (4.4–10.3) (1)	0.8 (.5–1.3) (13)	0.37 (.12–1.18)	…	0.47 (.22–.99)	.02
*Pneumocystis* pneumonia	2.8 (1.1–6.9) (10)	1.0 (.7–1.3) (4)	5.5 (1.4–20.1) (2)	0.7 (.1–4.0) (9)	0.13 (.03–.62)	…	0.33 (.10–1.13)	.02
Oral candidiasis	19.1 (13.0–27.3) (10)	2.3 (1.6–3.3) (4)	1.2 (.9–1.5) (1)	5.0 (3.2–7.7) (7)	0.09 (.03–.25)	…	0.20 (.08–.51)	<.001
Esophageal candidiasis	3.0 (1.7–5.3) (8)	0.2 (.1–.7) (1)	13.2 (9.7–17.4) (1)	1.0 (.5–2.0) (6)	0.31 (.06–1.64)	…	…	.15
Herpes zoster	9.4 (6.7–13.2) (7)	2.3 (1.6–3.3) (2)	…	4.3 (2.3–8.0) (7)	0.39 (.15–1.03)	…	…	.06
Herpes simplex/GUD	6.0 (2.7–12.8) (10)	1.2 (.2–7.7) (3)	5.7 (3.4–8.8) (1)	4.6 (1.8–11.3) (5)	0.18 (0–12.03)	…	0.58 (.04–7.50)	.63
Kaposi sarcoma	1.2 (.6–2.3) (14)	0.2 (0–1.5) (3)	2.0 (.4–8.5) (2)	0.5 (.3–.8) (6)	0.13 (.01–1.70)	…	0.77 (.11–5.20)	.26
Cerebral toxoplasmosis	1.1 (.6–2.1) (11)	0.6 (.2–1.7) (4)	11.0 (7.8–15.0) (1)	0.5 (.2–1.1) (6)	0.12 (.03–.57)	…	0.55 (.21–1.49)	.03
*Cryptosporidium* diarrhea	2.0 (.9–4.6) (6)	0.3 (0–2.9) (2)	…	0.5 (.2–1.3) (3)	…	…	…	NA
*Mycobacterium tuberculosis* (unspecified)	10.0 (8.7–11.5) (45)	4.2 (3.5–4.9) (33)	2.8 (1.2–6.5) (14)	6.8 (4.4–10.3) (22)	0.36 (.24–.55)	0.26 (.15–.45)	0.66 (.42–1.04)	<.001
Pulmonary tuberculosis	9.0 (6.8–11.8) (21)	3.5 (2.9–4.3) (15)	1.0 (.2–5.8) (3)	4.9 (3.1–7.7) (15)	0.38 (.22–.67)	0.13 (.04–.43)	0.52 (.52–.91)	<.001
Extrapulmonary tuberculosis	2.9 (2.0–4.1) (19)	1.2 (.9–1.7) (14)	0.2 (.1–.4) (1)	2.2 (1.4–3.3) (14)	0.43 (.25–.77)	…	0.61 (.33–1.11)	.02
Bacterial pneumonia	6.1 (4.3–8.4) (9)	0 (0–3.3) (1)	…	3.4 (1.0–11.1) (3)	…	…	…	NA
Bacteremia	5.3 (3.9–7.3) (5)	0.7 (.2–3.5) (2)	…	2.9 (.2–37.3) (2)	…	…	…	NA
Bacterial enteritis	5.3 (2.6–8.8) (6)	…	…	1.2 (.1–9.8) (3)	…	…	…	NA

Individual reports can be included in >1 ART category.

Abbreviations: ART, antiretroviral therapy; CI, confidence interval; GUD, genital ulcer disease; NA, numbers insufficient to perform analysis.

^a^ Reference is naive population, adjusted for region, ART status, and median follow-up time.

^b^ Determined by random-effects logistic meta-regression; ART categories with ≤3 studies were not included in the meta-regression analysis.

### Effect of ART on Incidence of OIs

During the first year of ART, the risk of all OIs declined to <2%, except for unspecified tuberculosis (4.2% [95% CI, 3.5%–4.9%]), PTB (3.5% [95% CI, 2.9%–4.3%]), herpes zoster (2.3% [95% CI, 1.6%–3.3%]), and oral candidiasis (2.3% [95% CI, 1.6%–3.4%]), which remained the most common OIs (Table 2 and Figure 3*B*).

Meta-regression analysis found that the greatest effect of ART was seen during the first year of treatment, and ranged from a 57%–91% reduction. There were few studies providing information on risk after the first year of ART except for unspecified tuberculosis and PTB. The magnitude of effect of ART during the first year was greatest for oral candidiasis (aOR, 0.09 [95% CI, .03–.25]), cerebral toxoplasmosis (aOR, 0.12 [95% CI, .03–.57]), and PCP (aOR, 0.13 [95% CI, .03–.62]) (Table 2). There was also a non–statistically significant reduction for cryptococcal meningitis (aOR, 0.37 [95% CI, .12–1.18]), herpes zoster (aOR, 0.39 [95% CI, .15–1.03]), Kaposi sarcoma (aOR, 0.13 [95% CI, .01–1.70]), esophageal candidiasis (aOR, 0.31 [95% CI, .06–1.64]), and GUD (aOR, 0.18 [95% CI, 0–12.03]). There was a 57%–64% reduction in risk of tuberculosis (unspecified: aOR, 0.36 [95% CI, .24–.55]; PTB: aOR, 0.38 [95% CI, .22–.67]; and EPTB: aOR, 0.43 [95% CI, .25–.77]). After the first year, there was a further reduction in risk for PTB (aOR, 0.13 [95% CI, .04–.43]) and unspecified tuberculosis (aOR, 0.26 [95% CI, .15–.45]). Summary risks for unspecified time on ART also showed a reduction in incidence for each OI, although this was less marked than during the first year of ART.

In the meta-regression analysis of the effect of region on incidence (Supplementary Appendix 3 and Supplementary Table 1), compared with sub-Saharan Africa, there was an increased risk of PCP in studies from Latin America (aOR, 10.58 [95% CI, 2.52–44.31]) and Asia (aOR, 9.88 [95% CI, 2.68–36.39]); and in Asia for toxoplasmosis (aOR, 7.46 [95% CI, 2.31–24.14]), cryptococcal meningitis (aOR, 2.71 [95% CI, 1.33–5.50]), and EPTB (aOR, 2.35 [95% CI, 1.31–4.23]). There were no significant regional differences for oral or esophageal candidiasis, herpes zoster, or GUD.

There was between-study heterogeneity in incidence of OIs across studies and regions, and the most important sources of heterogeneity were baseline CD4 count for cryptococcal meningitis (*I*^2^ = 56.4% for CD4 counts 200–499 cells/µL) and oral candidiasis (*I*^2^ = 54.9% for unspecified CD4 count), but no factor was identified that predominately explained the source of this heterogeneity. The use of CTX prophylaxis did not explain the heterogeneity in incidence of PCP or toxoplasmosis, where estimates of *I*^2^ were high for all categories of CTX exposure.

### Global Impact of ART Use on OIs Averted and Cost Savings

The use of ART before a decline in CD4 count to <200 cells/µL was estimated to have averted 857 828 OI cases (95% CI, 828 032–874 853) in 2013 (Table 3): 599 711 (95% CI, 595 974–629 673) in sub-Saharan Africa, 195 312 (95% CI, 194 096–205 071) in Asia, and 38 198 (95% CI, 37 962–40 109) in Latin America. The most common OIs, oral candidiasis (366 661 [95% CI, 356 835–368 546]) and tuberculosis (182 017 [95% CI, 173 392–190 468]), accounted for the greatest number of OIs averted. Estimated cost savings in OI treatment during 2013 were $46.7 million (95% CI, $43.8–$49.4 million) for 6 OIs with global estimates of treatment costs per case. Most savings from ART use were due to averted cases of tuberculosis, with an estimated $33.3 million (95% CI, $31.6–$34.8 million) in savings.

**Table 3.** Estimated Number of Opportunistic Infection Cases and Costs Averted During the First Year of Antiretroviral Therapy

Opportunistic Infection	No. of Cases Averted (95% CI)				Cost	
	Sub-Saharan Africa	Asia	LAC	Total	Cost per Case	Total Savings (95% CI)
*Cryptococcal* meningitis	15 970 (9722–21 716)	5201 (3166–7072)	1017 (619–1383)	21 766 (13 954–29 505)	$301.00	$6 551 455 ($4 201 751–$8 880 380)
*Pneumocystis* pneumonia	38 442 (16 227–38 457)	10 891 (5285–12 525)	2130 (1034–2450)	50 513 (36 439–54 028)	$53.97	$2 726 188 ($1 966 889–$2 915 872)
Oral candidiasis	258 035 (251 372–264 831)	84 036 (81 866–86 249)	16 436 (16 012–16 869)	366 661 (356 835–368 546)	$3.65	$1 338 313 ($1 302 460–$1 345 192)
Esophageal candidiasis	33 885 (27 776–40 777)	11 035 (9046–13 280)	2158 (1769–2597)	49 309 (40 301–57 887)	$18.79	$926 523 ($757 352–$1 087 692)
Herpes zoster	121 518 (116 659–128 096)	39 576 (37 993–41 718)	7740 (7431–8159)	168 948 (161 748–176 422)	$11.14	$1 882 079 ($1 801 885–$1 965 318)
Cerebral toxoplasmosis^a^	8812 (0–14 847)	2870 (0–4835)	561 (0–946)	18 614 (5642–21 225)	…	…
*Mycobacterium tuberculosis*	128 049 (121 905–134 494)	41 703 (39 702–43 802)	8156 (7765–8567)	182 017 (173 392–190 468)	$182.76	$33 265 448 ($31 689 493–$34 809 480)
Total (95% CI)	599 711 (595 974–629 673)	195 312 (194 096–205 071)	38 198 (37 962–40 109)	857 828 (828 032–874 853)		$46 690 006 ($43 777 338–$49 432 118)

Cases averted were calculated using Joint United Nations Programme on HIV/AIDS country estimates of the population in each region with a CD4 count <200 cells/µL in 2013, for opportunistic infections (OIs) with a *P* < .20 for evidence of an effect of antiretroviral therapy (ART). Uncertainty ranges were estimated from 1000 Monte Carlo draws of the difference in estimates of risk between early ART and ART-naive populations, assuming a normal distribution. The total for all opportunistic infections (OIs) is the median of the sum of 1000 random draws for each OI, and therefore differs from the sum of the medians of number of cases averted by region.

Abbreviations: CI, confidence interval; LAC, Latin America and the Caribbean.

^a^ Global costs per case were not available for cerebral toxoplasmosis.

## DISCUSSION

This systematic review and meta-analysis is the most comprehensive assessment of the global and regional incidence and effect of ART for 15 OIs, based on an analysis of almost 500 000 HIV-infected adults. Overall, there was a substantial reduction in risk during the first year of ART, ranging from 57% to 91%, which was greatest for oral candidiasis, toxoplasmosis, and PCP. The magnitude of effect of ART is more pronounced than that observed in HICs, which is consistent with evidence that levels of treatment adherence and immune recovery in LMICs are comparable to those in HICs [19–28]. A similar reduction in incidence of OIs has been observed in a companion meta-analysis of children in LMICs [29], although the incidence of bacterial pneumonia was substantially higher in children, whereas herpes zoster was more frequent in ART-naive adults.

We had insufficient studies for a robust analysis of the effect of ART on bacterial infections, but showed some effect of ART on bacterial pneumonia. Data from other studies show that the level of immune suppression has little effect on the acquisition of bacterial infections [30, 31]. Overall, it is likely that some of the reduction in risk with ART was due to increasing CTX chemoprophylaxis [26]. This highlights the importance of providing CTX to all HIV-infected adults and children in LMICs.

There were few studies providing data on longer-term effects of ART, other than for unspecified tuberculosis and PTB. The effect of ART was greater after 1 year, and is consistent with other studies showing a progressive time-dependent reduction in risk over the first 2–3 years of ART [32, 33]. This can be explained by the occurrence of tuberculosis across a wide range of CD4 counts, with less of a protective effect of early immune restoration, and because of the significant rate of unmasking immune reconstitution inflammatory syndrome (IRIS) in the first months after ART initiation. It is likely that further stratification within the first year of therapy would have revealed variations in the effect of ART for all OIs, with an increase in diagnoses seen in the first 3 months and then a gradual decline in incidence. This could explain why duration of follow-up did not seem to impact study heterogeneity, as most cases occur early during ART.

We were limited in our ability to draw definitive conclusions on the importance of regional differences, due to some imprecision in the estimates, as well as variability in diagnostic methods and inconsistency in ascertainment of cases. The low incidence of PCP and other OIs in sub-Saharan Africa may be attributable to limited diagnostic resources and capacity, rather than true lower endemicity [34], and is supported by the lack of regional variation in those OIs that are predominantly diagnosed clinically.

Our analyses also provide a valuable approximation of number of OIs averted, and potential cost savings of $47 million per annum, from initiating ART before the CD4 count declines to <200 cells/µL. These estimates likely underestimate the full savings as they do not capture variation in treatment costs across different countries or include the diagnostic costs. Although the costs of ART tend to be higher than the costs of treating OIs, the savings from averting these OIs are a substantial additional benefit of ART.

As with other meta-analyses of observational studies, there was high heterogeneity due to inclusion of diverse populations with varying times of follow-up, variability in reporting of CTX, ART regimen, and duration of ART, and lack of data on important confounders such as baseline CD4 count. Few studies distinguished between presumptive and conclusive OI diagnoses. There were also significant gaps in data from regions such as Latin America, a predominance of data from sub-Saharan Africa, and large cohorts in Uganda, Cote D'Ivoire, and South Africa, and from older cohorts with more advanced immunodeficiency at ART initiation, which precluded an evaluation of the impact of initiating ART at higher CD4 counts.

In conclusion, the profound effect of ART on the incidence of most HIV-related OIs is the key reason for the observed global decline in HIV-related mortality, and highlights the continued priority of expanding ART access. It is estimated that expanding ART access to all people living with HIV will avert 21 million AIDS-related deaths by 2030. The WHO now recommends ART for all HIV-infected persons [35], based on the Strategic Timing of Antiretroviral Treatment and Trial of Early Antiretrovirals and Isoniazid Preventive Therapy in Africa trials findings of a major reduction in AIDS and non-AIDS-related morbidity and mortality with ART initiation at a CD4 count >500 cells/µL [36, 37], as well as evidence of a marked impact on HIV transmission [32]. A significant proportion of HIV-infected persons continue to present with advanced disease, and so other measures to reduce mortality remain important. This includes prompt initiation of CTX prophylaxis, screening for tuberculosis and cryptococcal disease, and use of isoniazid and fluconazole chemoprophylaxis in those with advanced disease.

## Supplementary Data

Supplementary materials are available at http://cid.oxfordjournals.org. Consisting of data provided by the author to benefit the reader, the posted materials are not copyedited and are the sole responsibility of the author, so questions or comments should be addressed to the author.

Supplementary Data
