# Supplementary material for: Incidence of Opportunistic Infections and the Impact of Antiretroviral Therapy Among HIV-Infected Adults in Low- and Middle-Income Countries: A Systematic Review and Meta-analysis
Source: Clin Infect Dis. 2016 Mar 6;62(12):1595–603. doi: 10.1093/cid/ciw125 (PMC4885646; doi:10.1093/cid/ciw125)
Supplement: Supplementary Data [file supp_62_12_1595__index.html]

Incidence of Opportunistic Infections and the impact of Antiretroviral Therapy among HIV-Infected Adults in Low and Middle Income Countries: a Systematic Review and Meta-analysis — Incidence of Opportunistic Infections and the Impact of Antiretroviral Therapy Among HIV-Infected Adults in Low- and Middle-Income Countries: A Systematic Review and Meta-analysis — Incidence of Opportunistic Infections and the Impact of Antiretroviral Therapy Among HIV-Infected Adults in Low- and Middle-Income Countries: A Systematic Review and Meta-analysis — Supplementary Data 

# Incidence of Opportunistic Infections and the Impact of Antiretroviral Therapy Among HIV-Infected Adults in Low- and Middle-Income Countries: A Systematic Review and Meta-analysis

## Supplementary Data

Supplementary Data

- Supplementary Data - Docx file
