# Supplementary material for: Incidence of Opportunistic Infections and the Impact of Antiretroviral Therapy Among HIV-Infected Adults in Low- and Middle-Income Countries: A Systematic Review and Meta-analysis
Source: Clin Infect Dis. 2016 Mar 6;62(12):1595–603. doi: 10.1093/cid/ciw125 (PMC4885646; doi:10.1093/cid/ciw125)
Supplement: Supplementary Data [file supp_ciw125_ciw125supp.docx]

**Appendices**

**Appendix 1: Search Strategy**

The following search strings/criteria were used on November 18, 2013. Searches of reference lists were also undertaken for all relevant citations.

**A. Search string for PubMed/Medline**

The following search string was limited to references published after January 1, 1990:

(“HIV”[MeSH] OR “human immunodeficiency virus”[text word] OR “[Acquired Immunodeficiency Syndrome](http://www.ncbi.nlm.nih.gov/mesh/68000163)”[MeSH] OR “HIV/AIDS”[text word] OR “HIV”[text word] OR “AIDS”[text word] OR “*Hiv-1”[MeSH]) AND (“[AIDS-Related Opportunistic Infections](http://www.ncbi.nlm.nih.gov/mesh/68017088)”[MeSH] OR “AIDS-Related Opportunistic Infections/*etiology”[MeSH] OR “[Opportunistic Infections](http://www.ncbi.nlm.nih.gov/mesh/68009894)”[MeSH] OR “opportunistic infection”[text word] OR “opportunistic”[text word] OR “Acquired Immunodeficiency Syndrome/*epidemiology”[MeSH] OR “[Sarcoma, Kaposi](http://www.ncbi.nlm.nih.gov/mesh/68012514)”[MeSH] OR “Kaposi*”[text word] OR “Sarcoma, Kaposi/epidemiology”[MeSH] OR “sarcoma”[text word] OR “KS”[text word] OR “[Toxoplasmosis, Cerebral](http://www.ncbi.nlm.nih.gov/mesh/68016781)”[MeSH] OR “toxoplasm*”[text word] OR “toxoplasmosis”[MeSH] OR “[Toxoplasmosis, Ocular](http://www.ncbi.nlm.nih.gov/mesh/68014126)”[MeSH] OR “[Cytomegalovirus Retinitis](http://www.ncbi.nlm.nih.gov/mesh/68017726)”[MeSH] OR “[Cytomegalovirus](http://www.ncbi.nlm.nih.gov/mesh/68003587)”[MeSH] OR “CMV”[text word] OR “Cytomegalovirus”[text word] OR “[Pneumonia, Pneumocystis](http://www.ncbi.nlm.nih.gov/mesh/68011020)”[MeSH] OR “[Pneumocystis jirovecii](http://www.ncbi.nlm.nih.gov/mesh/68045382)”[MeSH] OR “[Pneumocystis carinii](http://www.ncbi.nlm.nih.gov/mesh/68045363)”[MeSH] OR “Pneumocystis”[text word] OR “PCP”[text word] OR “PJP”[text word] OR “Cryptococcus”[MeSH] OR “[Cryptococcus neoformans](http://www.ncbi.nlm.nih.gov/mesh/68003455)”[MeSH] OR “Cryptococcus”[text word] OR “Cryptococcosis”[text word] OR “Meningitis, Cryptococcal”[MeSH] OR “*Meningitis”*[MeSH] OR “[Herpes Simplex](http://www.ncbi.nlm.nih.gov/mesh/68006561)”[MeSH] OR “[Herpes Genitalis](http://www.ncbi.nlm.nih.gov/mesh/68006558)”[MeSH] OR “herpes”[text word] OR “herpes simplex”[text word] OR “HSV*”[text word] OR “genital ulcer*”[text word] OR “Herpes zoster”[MeSH] OR “varicella zoster”[text word] OR “zoster”[text word] OR “shingle*”[text word] OR “rash”[text word] OR “[Diarrhea](http://www.ncbi.nlm.nih.gov/mesh/68003967)”[MeSH] OR “[Cryptosporidiosis](http://www.ncbi.nlm.nih.gov/mesh/68003457)”[MeSH] OR “[Enteritis](http://www.ncbi.nlm.nih.gov/mesh/68004751)”[MeSH] OR “[Diarrhea](http://www.ncbi.nlm.nih.gov/mesh/68003967)”[text words] OR “Cryptosporidi*”[text words] OR “[Enteritis](http://www.ncbi.nlm.nih.gov/mesh/68004751)”[text words] OR “[Tuberculosis](http://www.ncbi.nlm.nih.gov/mesh/68014376)”[MeSH] OR “TB”[text word] OR “Tuberculosis”[text word] OR “[Mycobacterium tuberculosis](http://www.ncbi.nlm.nih.gov/mesh/68009169)”[MeSH] OR “Mycobacterium tuberculosis”[text words] OR “Candida”[MeSH] OR “[Candidiasis](http://www.ncbi.nlm.nih.gov/mesh/68002177)”[MeSH] OR “[Candidiasis, Oral](http://www.ncbi.nlm.nih.gov/mesh/68002180)”[MeSH] OR “Candida”[text word] OR “Candidiasis”[text word] OR “thrush”[text word] OR “Pneumonia”[text word] OR “[Pneumonia](http://www.ncbi.nlm.nih.gov/mesh/68011014)”[MeSH] OR “[Pneumonia, Bacterial](http://www.ncbi.nlm.nih.gov/mesh/68018410)”[MeSH] OR “Pneumonia”[text word] OR “[Streptococcus pneumoniae](http://www.ncbi.nlm.nih.gov/mesh/68013296)”[MeSH] OR “Streptococcus pneumoniae”[text word] OR “Streptococc*”[text word] OR “[Pneumococcal Infections](http://www.ncbi.nlm.nih.gov/mesh/68011008)”[MeSH] OR “bacteremia”[text word] or “bacteraemia”[text word] OR “[Salmonella](http://www.ncbi.nlm.nih.gov/mesh/68012475)”[MeSH] OR “Salmonella”[text word] OR “sepsis”[text word] OR “[Sepsis](http://www.ncbi.nlm.nih.gov/mesh/68018805)”[MeSH] OR “[Bacteremia](http://www.ncbi.nlm.nih.gov/mesh/68016470)”[MeSH] NOT (“Hepatitis C”[MeSH] OR “Hepatitis B”[MeSH]) ) AND (“Asia”[text word] OR “Asia”[MeSH] OR “Africa”[text word] OR “Africa”[MeSH] OR “South America”[text word] OR “Latin America”[text word] OR “Caribbean”[text word] OR “Caribbean region”[MeSH] OR “developing countries”[MeSH] OR “Brazil”[MeSH] OR “Uganda”[MeSH] OR “resource limited setting*”[text word] OR “low resource” [text word] OR “low income”[text word] OR “middle income”[text word] OR “low-income”[text word] NOT (“United States”[MeSH] OR “North America”[MeSH] OR “Australia”[MeSH] OR “Europe”[MeSH])) AND “Humans”[MeSH] AND (“Adult”[MeSH] OR “adult”[text word] OR “adults”[text word] OR “Middle Aged”[MeSH]) AND ((Journal Article[pt]OR Letter[pt]) NOT “Case Reports”[Publication type]) AND (“Cohort studies”[MeSH] OR “Incidence”[MeSH] OR “incidence”[text word] OR “cohort”[text word] OR “Risk”[MeSH] OR “risk”[text word] OR “rate*”[text word] OR “Epidemiology”[MeSH] OR “Proportional Hazards Models”[MeSH] OR “Hospital mortality”[MeSH] OR “longitudinal”[text word]OR “Cross-Sectional Studies”[MeSH]OR “Prospective Studies”[MeSH]) AND "1990"[Entrez Date] : "3000"[Entrez Date] AND (English[Language] OR French[Language] OR Spanish[Language] OR Portuguese[Language])

**B. Search string for Ovid databases EMBASE, Global Health and Medline, searched together**

This search was combined using the Ovid database interface.

1. adult*.mp. [mp=ti, ab, sh, hw, tn, ot, dm, mf, bt, nm, ui]

2. limit 1 to abstracts

3. limit 2 to (english or french or portuguese or spanish)

4. limit 3 to yr="1990 -Current"

5. ("HIV" or "human immunodeficiency virus" or "Acquired Immunodeficiency Syndrome" or "HIV/AIDS" or "HIV" or "AIDS" or "HIV-1").mp. [mp=ti, ab, sh, hw, tn, ot, dm, mf, bt, nm, ui]

6. limit 5 to abstracts

7. limit 6 to (english or french or portuguese or spanish)

8. limit 7 to yr="1990 -Current"

9. ("AIDS-Related Opportunistic Infections" or "opportunistic" or "opportunistic infection" or tuberculosis or "mycobacterium tuberculosis" or "TB" or cryptospori* or cryptococc* or streptococc* or pneumococc* or pneumocyst* or "PCP" or "PJP" or meningitis or pneumonia or sepsis or enteritis or toxoplasm* or "herpes zoster" or cytomegalovirus or CMV or candid* or thrush or Kaposi or "herpes simplex" or herpes or "HSV" or "herpes virus" or "varicella zoster" or "zoster" or "shingle" or "bacteremia" or "bacteraemia").mp. [mp=ti, ab, sh, hw, tn, ot, dm, mf, bt, nm, ui]

10. limit 9 to abstracts

11. limit 10 to (english or french or portuguese or spanish)

12. limit 11 to yr="1990 -Current"

13. (“Asia” or “Africa” or "Latin America" or "South America" or “developing country” or “low resource” or "low income” or “middle income” or “resource limited” or “Caribbean”).mp. [mp=ti, ab, sh, hw, tn, ot, dm, mf, bt, nm, ui]

14. limit 13 to abstracts

15. limit 14 to (english or french or portuguese or spanish)

16. limit 15 to yr="1990 -Current"

17. 4 and 8 and 12 and 16

18. remove duplicates from 17

**C. Search string for Web of Science**

The Web of Science search was conducted from 1990 to November 2013 using the Science Citation Index Expanded and the Conference Proceedings Citation Index for Science and Social Science and Humanities. Reviews were excluded from inclusion but reference lists were examined. The following lines were searched under all topics.

adult*

AND "HIV" or "human immunodeficiency virus" or "Acquired Immunodeficiency Syndrome" or "HIV/AIDS" or "HIV" or "AIDS" or "HIV-1

AND "AIDS-Related Opportunistic Infections" or "opportunistic" or "opportunistic infection" or tuberculosis or "mycobacterium tuberculosis" or "TB" or cryptospori* or cryptococc* or streptococc* or pneumococc* or pneumocyst* or "PCP" or "PJP" or meningitis or pneumonia or sepsis or enteritis or toxoplasm* or "herpes zoster" or cytomegalovirus or CMV or candid* or thrush or Kaposi or "herpes simplex" or herpes or "HSV" or "herpes virus" or "varicella zoster" or "zoster" or "shingle" or "bacteremia" or "bacteraemia”

AND “Asia” or “Africa” or "Latin America" or "South America" or “developing country” or “low resource” or "low income” or “middle income” or “resource limited” or “Caribbean”

**D. Search string for LILACS**

The above string was adapted for the LILACS user interface using each term as “words”. Titles were reviewed and exported as txt files.

adult AND HIV AND opportunistic OR tuberculosis OR AIDS OR "mycobacterium tuberculosis" OR TB OR cryptosporium OR cryptococcus OR streptococcus OR pneumococcus OR pneumocystis OR "PCP" OR "PJP" OR meningitis OR pneumonia OR sepsis OR enteritis OR toxoplasma OR "herpes zoster" OR candida OR thrush OR Kaposi OR "herpes simplex" OR herpes OR "HSV" OR "herpes virus" OR "varicella zoster" OR "zoster" OR "shingle" OR "bacteremia" OR "bacteraemia”

**E. Search string for CINAHL**

The following string was used for the nursing database CINAHL without selecting fields, with limiters to region and age groups:

"HIV" or "human immunodeficiency virus" or "Acquired Immunodeficiency Syndrome" or "HIV/AIDS" or "HIV" or "AIDS" or "HIV-1

AND

AIDS-Related Opportunistic Infections" or "opportunistic" or "opportunistic infection" or tuberculosis or "mycobacterium tuberculosis" or "TB" or cryptospori* or cryptococc* or streptococc* or pneumococc* or pneumocyst* or "PCP" or "PJP" or meningitis or pneumonia or sepsis or enteritis or toxoplasm* or "herpes zoster" or cytomegalovirus or CMV or candid* or thrush or Kaposi or "herpes simplex" or herpes or "HSV" or "herpes virus" or "varicella zoster" or "zoster" or "shingle" or "bacteremia" or "bacteraemia

**Limiters:** Exclude Medline records; Human; Publication Type: all; Sex: all; Age groups: adult, middle aged, and aged; Geographic Subset: Asia, Africa, Middle East, Mexico and Central/South America; Languages: English, Spanish, French, and Portuguese.

**F. Cochrane Library including Cochrane database of systematic reviews, DARE, Cochrane Central register of controlled trials (CENTRAL), Cochrane Methodology register, HTA and NHS EED**

Due to limited search functionality, Cochrane was searched using the term HIV and then AIDS. All identified reference lists were then reviewed manually to verify that our search had found all relevant articles.

**G. Search of the grey literature**

An initial review of the grey literature was conducted using the following string in Google scholar, and by searching Ministry of Health websites, clinical trials registries and who.int.

*adult AND HIV AND (opportunistic OR tuberculosis OR AIDS OR "mycobacterium tuberculosis" OR TB OR cryptosporidium OR cryptococcus OR streptococcus OR pneumococcus OR pneumocystis OR "PCP" OR "PJP" OR meningitis OR pneumonia OR sepsis OR enteritis OR toxoplasma)*

Although the search identified large numbers of publications including guidelines, initial review indicated that they were unsuitable due to lack of peer revision, no disaggregation by type of OI, no descriptions of diagnostic criteria or laboratory methodology, and few reports providing data allowing calculation of risk. It was therefore decided that this topic was unsuitable for grey literature inclusion.

**H. Conference abstracts**

The Conference on Retroviruses and Opportunistic Infections website was searched for abstracts using the following terms:

*opportunistic OR tuberculosis OR AIDS OR "mycobacterium tuberculosis" OR TB OR cryptosporidium OR cryptococcus OR streptococcus OR pneumococcus OR pneumocystis OR "PCP" OR "PJP" OR meningitis OR pneumonia OR sepsis OR enteritis OR toxoplasma*

The search was initially conducted in January 2011 and included abstracts from 1993 to 2010. After that, the website no longer held abstracts and we were unable to update our search for 2011-2013. Other conference abstracts were identified using Web of Science and the above databases.

**Appendix 2: Study Methodology**

**Data management**

All records were saved into an EndNote X5 file for merging with duplicate removal. This was done mechanically and manually by title. Conference abstracts were manually sorted and pdfs or posters reviewed if available. Relevant conference titles and authors were searched on Google scholar and Pubmed to see if more recent publications on the topic had been published, in which case this was used.

Data was extracted from full records into an excel file and included: a) year of publication, b) years of study, c) city and country, d) name of clinic and/or cohort, e) population, f) baseline median or mean CD4 counts and IQR or SD, g) study design, h) sampling strategy, i)sample size, j) proportion female, k) proportion on ART, l) time on ART (months), m) incidence rate per 100 person-years, n) incidence risk, o) CTX status (see below), p) methods of diagnosis, q) median follow-up time and range, and r ) study limitations. All data was double extracted and compared between 2 co-authors.

**Inclusion and exclusion criteria**

**Study design**

All study designs including experimental and observational data were included if they presented quantitative outcomes. Studies which did not include primary data or with less than 50 participants were excluded. Quasi-experimental studies were not immediately excluded but usually did not provide a reliable denominator for calculation of risk. Studies were excluded from the analysis of TB or cryptococcal/candidal disease if they reported the use of isoniazid or anti-fungal prophylaxis respectively.

**Definitions**

1. **HIV**: An adult living with HIV is a person aged 18 and older who has tested positive for antibodies to HIV-1. Due to potential variation in ART outcomes, pregnant women were not included, but all other population groups were.

2. **ART status:** The proportion of adults with HIV taking antiretrovirals who have a defined outcome allowing the calculation of risk. Studies where the proportion on ART was not available were excluded.

*a. Naïve:* included patients not taking ART, populations where ART is not commonly available, or where the proportion on ART is less than 10%.

b. *Prescribed:* included patients taking ART, and populations where the described proportion on ART is greater than 80%.

3. **Time on ART:**

a. *First year of ART*: The number of OIs per population during the first year of ART is provided. The earliest estimate within the first year was used.

b. *After the first year of ART*: The number of OIs per population after one year of ART is provided. If time on ART was stratified after one year, the period presenting an overall estimate for greater than one year was used.

c. *Unspecified ART*: The number of OIs per population on ART is not provided by duration of ART. This category included those with no information on duration or where the duration spanned the two categories were included in this category.

4. **Cotrimoxazole status:** Patients were considered to be on cotrimoxazole if explicitly stated in the text, including where they limit cotrimoxazole to patients with a CD4<200 cells/μl. Patients were also assumed to be on cotrimoxazole if cohorts had stated in previous papers that they had introduced it, or if national guidelines encouraged it and the study site stated that they followed national guidelines.

5. **CD4 count**: The median or mean baseline CD4 count for the population was used in the analysis of heterogeneity. In reports where it was not provided, it was classified as unknown. Where results were stratified by CD4 count, a summary incident risk was calculated for the overall group and a median CD4 count was estimated.

6. **Definitions of Outcomes:**

All studies which used presumptive or definitive methods of diagnosis were included. Studies where methods of diagnosis were not described were included but were classified as an indeterminate method of diagnosis in the meta-regression analysis.

**a. Cryptococcosis:** Cryptococcal disease included studies where it was described as meningitis, and invasive or extra-pulmonary cryptococcosis. Cryptococcal pneumonia was excluded. Cryptococcal diseases were defined by the use of CDC diagnostic criteria or laboratory testing such as cryptococcal antigens, or india ink.

b. ***Pneumocystis jiroveci* pneumonia:** This included conditions described as *Pneumocystis carinii* pneumonia and PCP or PJP. Studies using presumptive methods such as dyspnea, suspicious infiltrates on chest x-rays and response to appropriate therapy were included. Studies describing extra-pulmonary pneumocystosis exclusively were excluded.

c. **Candidiasis**: Candidal disease was classified as oral or oesophageal candidiasis and analysed separately. Oesophageal candidiasis included cases with oral candidiasis as part of the diagnostic criteria but oral candidiasis cases excluded cases defined as oesophageal. Cases described as candidiasis of the bronchi, trachea or lungs were excluded.

d. **Herpes zoster**: Included cases were described as herpes zoster, zoster, recurrent varicella zoster, and shingles. Studies were not excluded if they did not specify recurrent episodes or more than one dermatome.

e. **Genital herpes simplex or ulcer disease:** Included cases were described as genital ulcer disease, with confirmation of herpes simplex etiology by laboratory testing in few studies. Studies conducted in Sexually Transmitted Infection (STI) clinics where participants were at higher risk or attending due to symptoms were excluded.

f. **Kaposi’s sarcoma:** Included cases were described as newly diagnosed Kaposi’s sarcoma by clinical suspicion and occasional histological confirmation. Studies which did not specify whether cases were newly diagnosed or recurrent were excluded.

g. **Toxoplasmosis:** Included cases were described as cerebral toxoplasmosis, encephalitis, central nervous system toxoplasmosis and toxoplasmosis of the brain. Cases were diagnosed using clinical response to treatment and/or imaging studies.

h. **Cryptosporidium diarrhoea:** Included cases were described as cryptosporidiosis, with chronic diarrhoea of greater than 1 month’s duration. The majority of studies confirmed the aetiology of the chronic diarrhea with Ziehl-Neelsen staining but studies based just on clinical response were included.

i. ***Mycobacterium tuberculosis***: Cases of tuberculosis were classified as pulmonary, extra-pulmonary or unspecified/any site. Unspecified tuberculosis included the overall number of tuberculosis cases, combining pulmonary and extra-pulmonary cases to provide a summary estimate. The vast majority of studies used sputum AFB staining and/or mycobacterial culture for diagnosis. For those studies without laboratory confirmation, a clinical case definition was used, usually cited as conforming to CDC guidelines.

j. **Bacterial pneumonia, bacteraemia and enteritis:** Cases of bacterial disease were included if they specified the type of disease and location, and, in the case of bacteraemia, if they were described as ‘isolate bacteraemia’. Pneumonia cases were not limited to recurrent pneumonia. All cases were diagnosed by culture.

**Appendix 3: Supplementary results**

**I. figures showing incidence and 95% CI for each OI among ART naïve, first year of ART, After first year of ART or ART time unspecified, and by region**

***Supplementary figure* 1a: *Cryptococcal* meningitis**

*
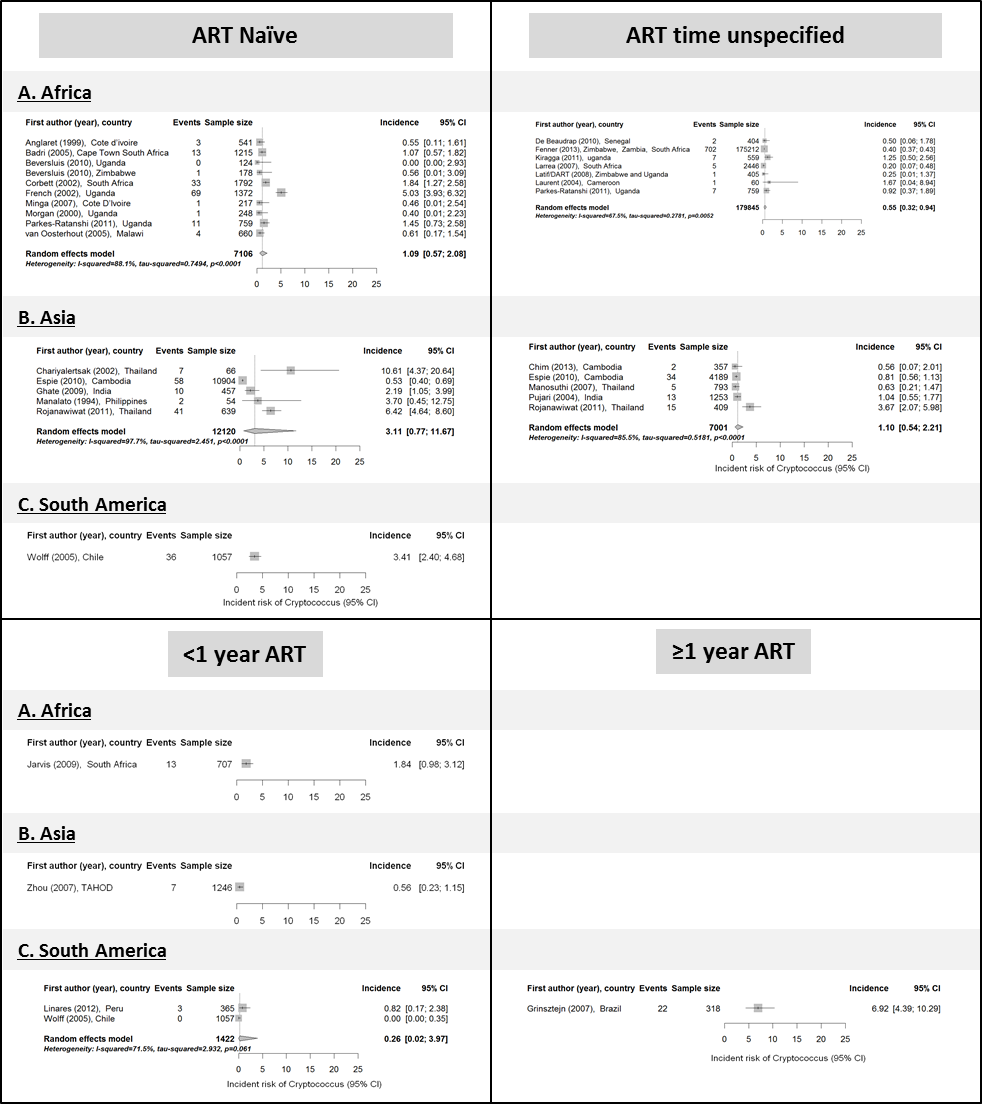
*

***Supplementary figure 1*b: *Pneumocystis jiroveci* pneumonia**

*
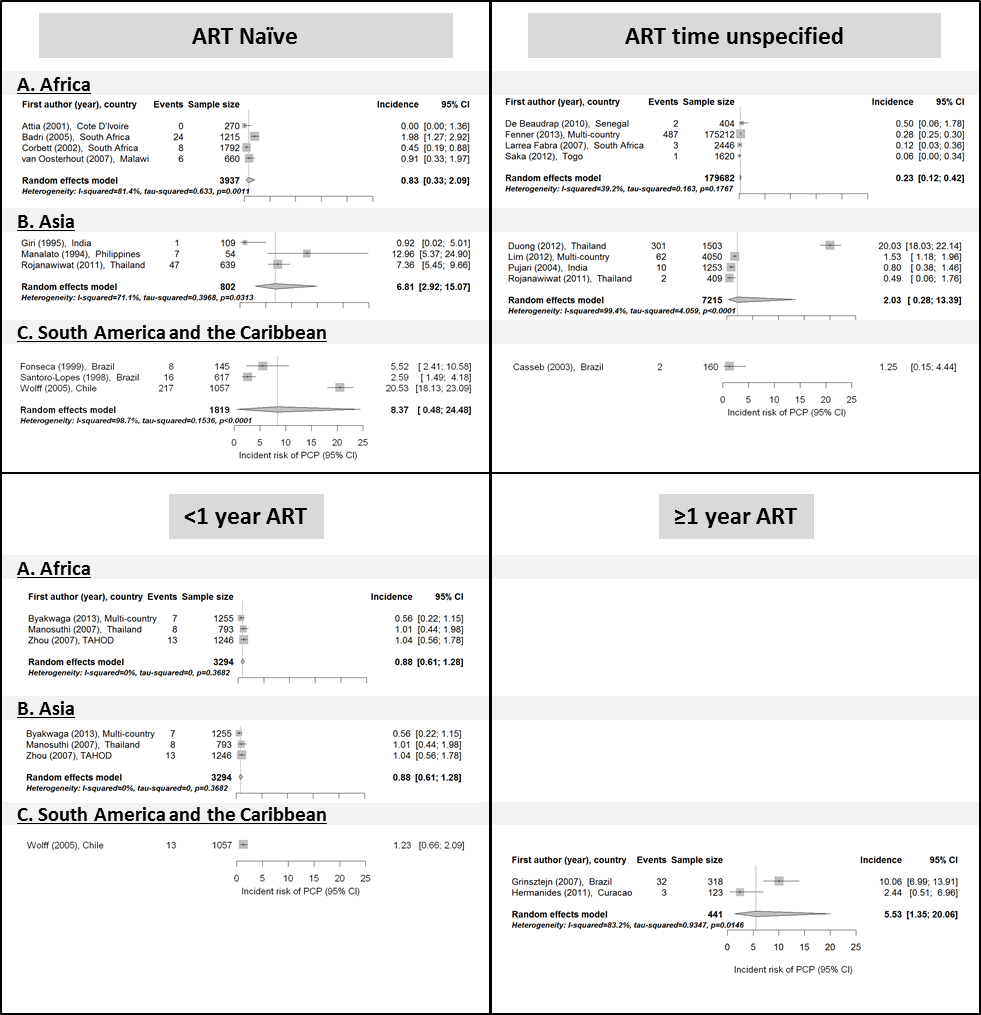
*

***Supplementary figure 1*c: Oral candidiasis**


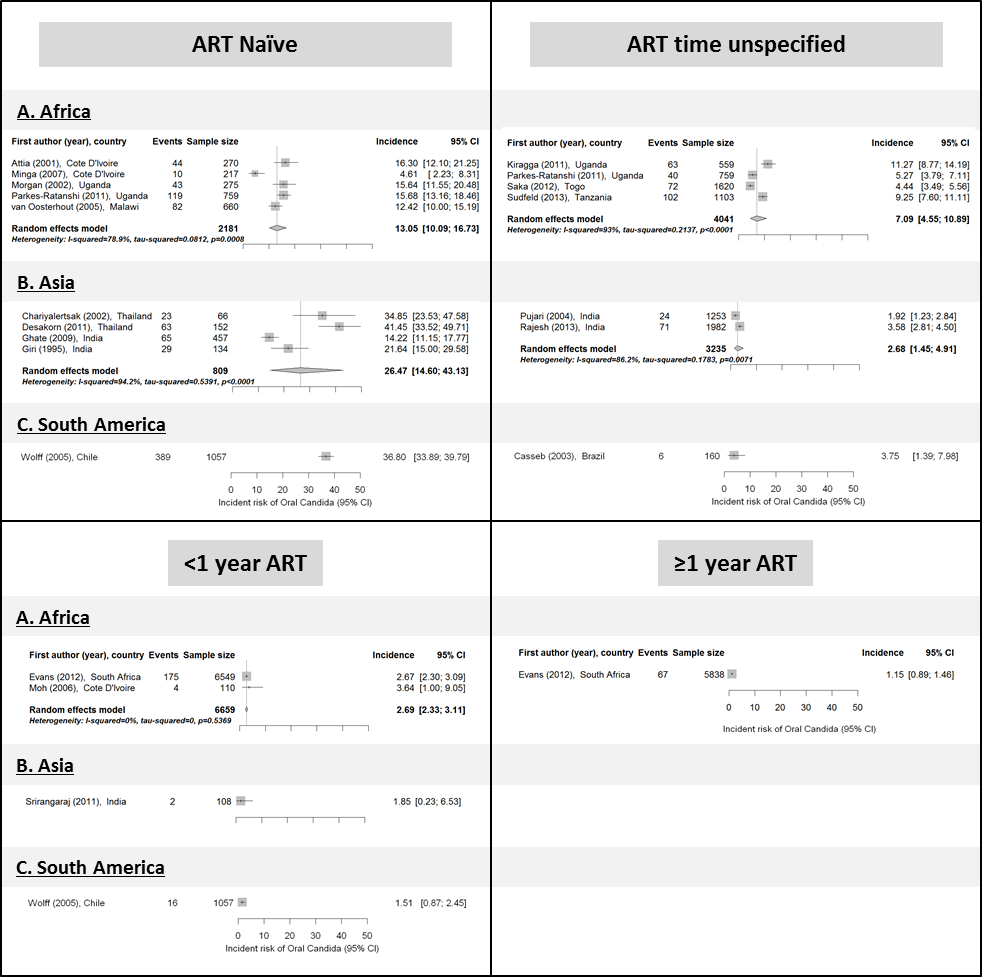


***Supplementary figure* 1d: Oesophageal candidiasis**


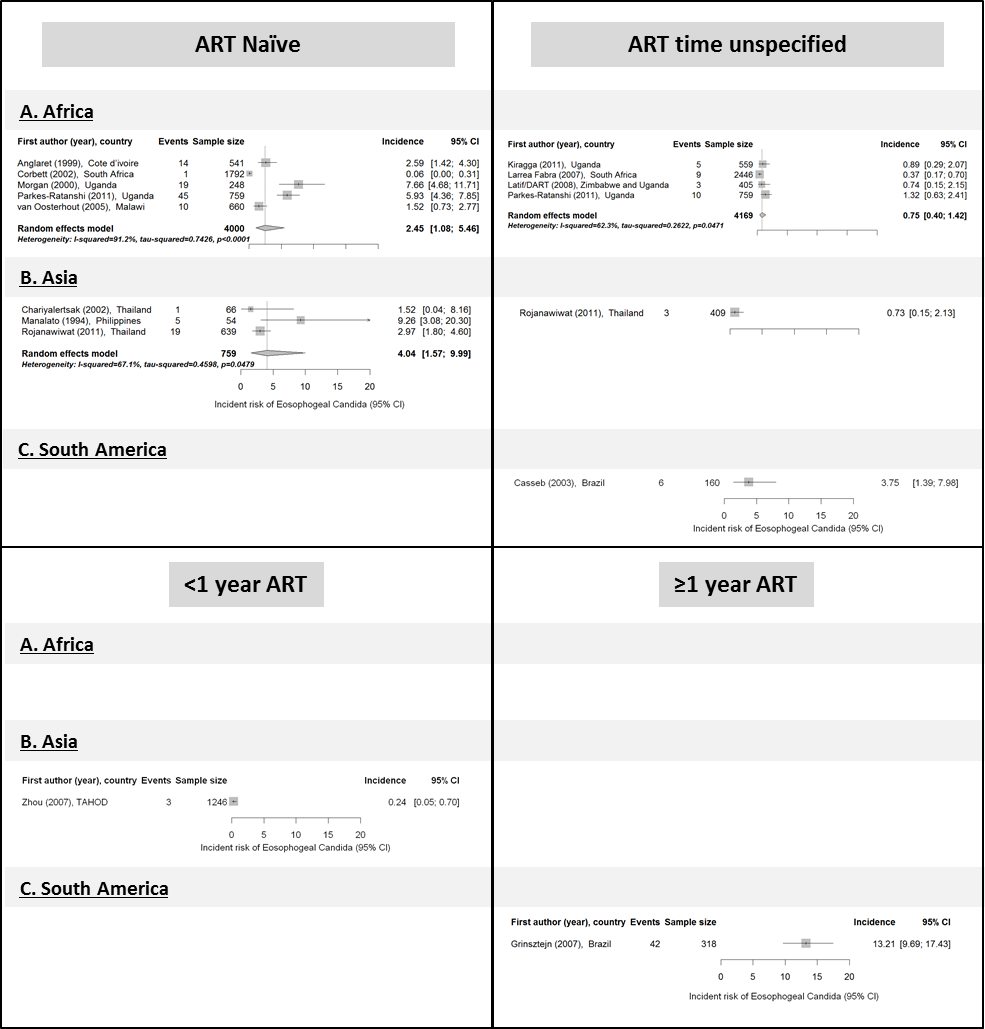


***Supplementary figure* 1e: Herpes Zoster or Shingles**


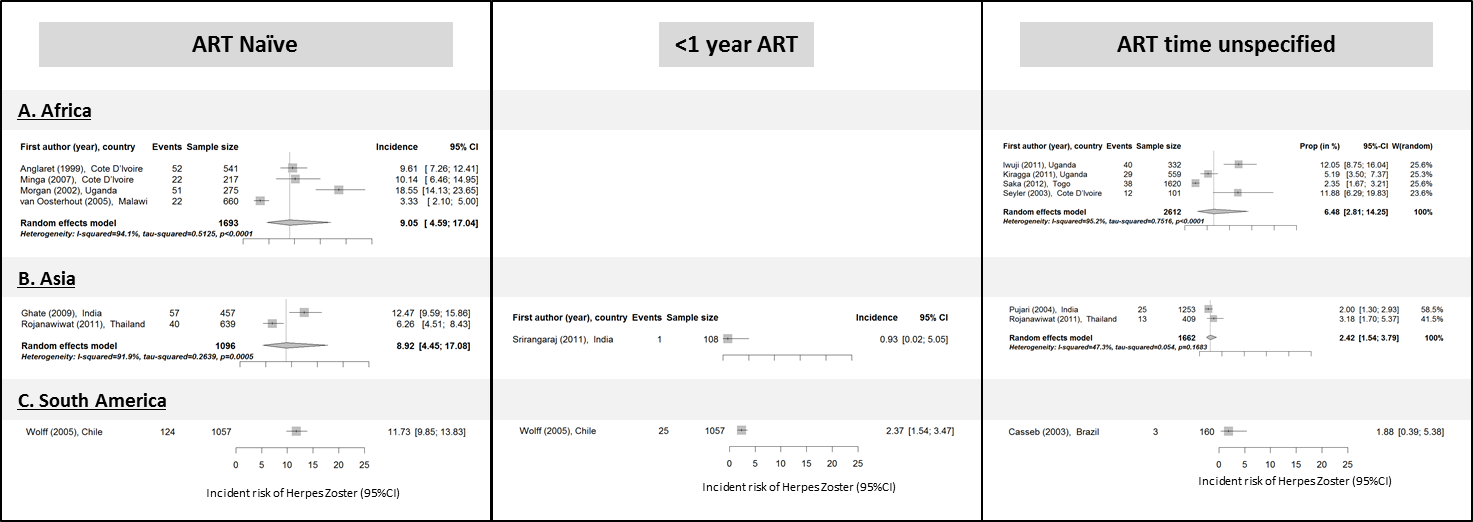


***Supplementary figure* 1f: *Herpes simplex virus*/genital ulcer disease**

*
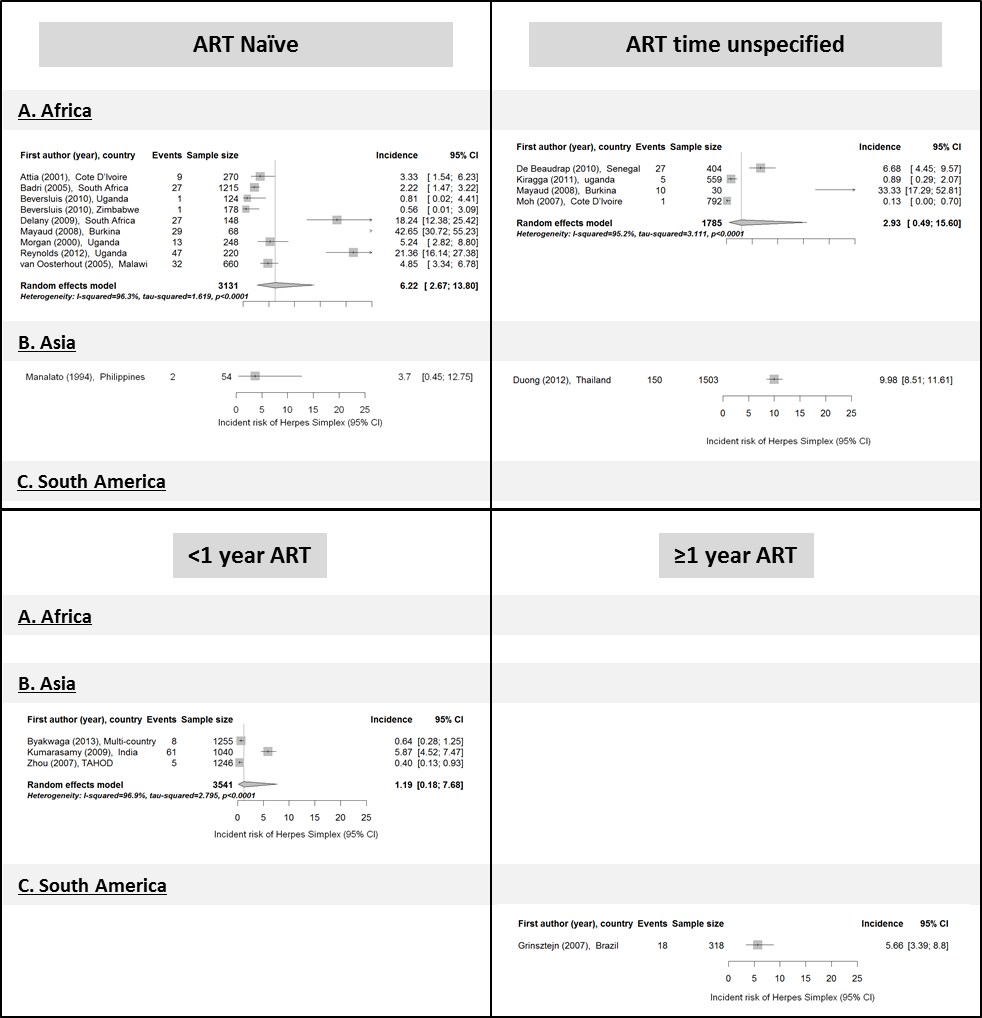
*

***Supplementary figure 1*g: Kaposi’s sarcoma**

*
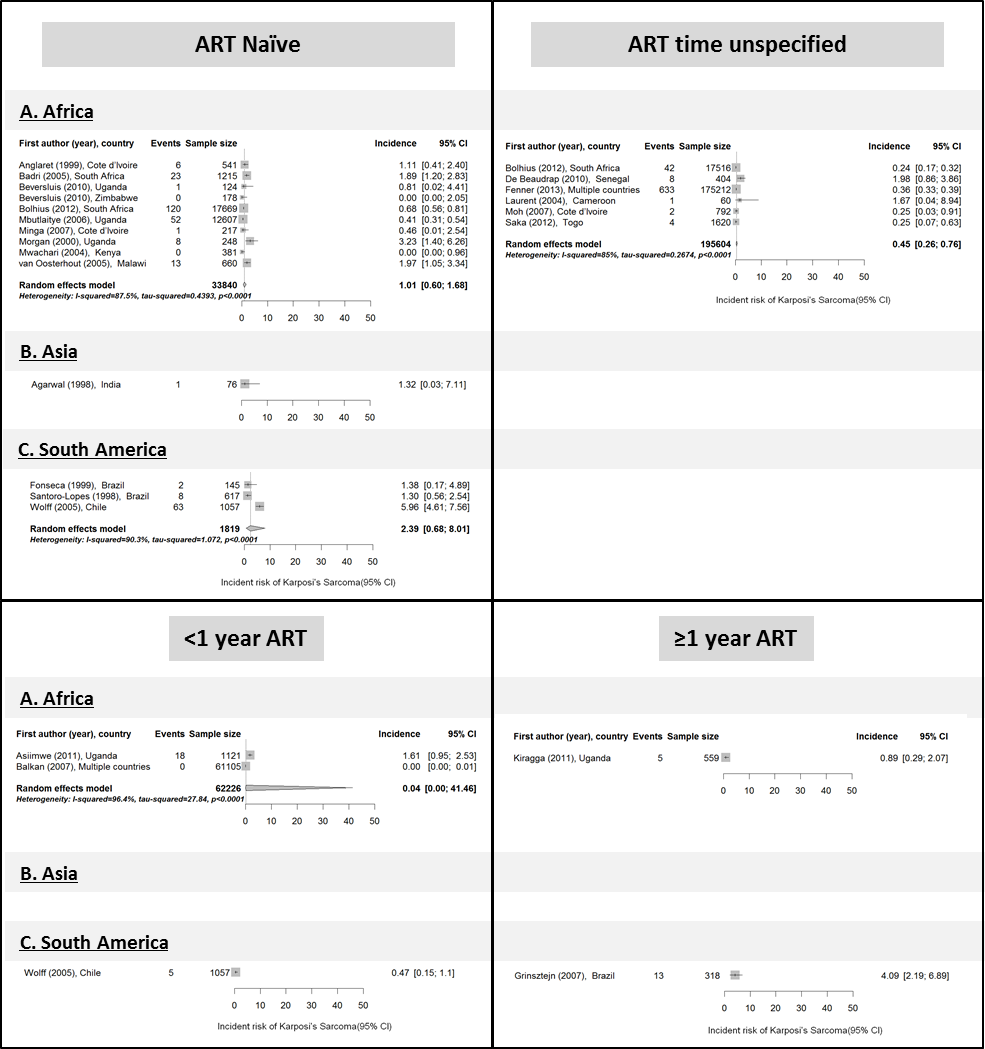
*

***Supplementary figure* 1h: Cerebral toxoplasmosis**

*
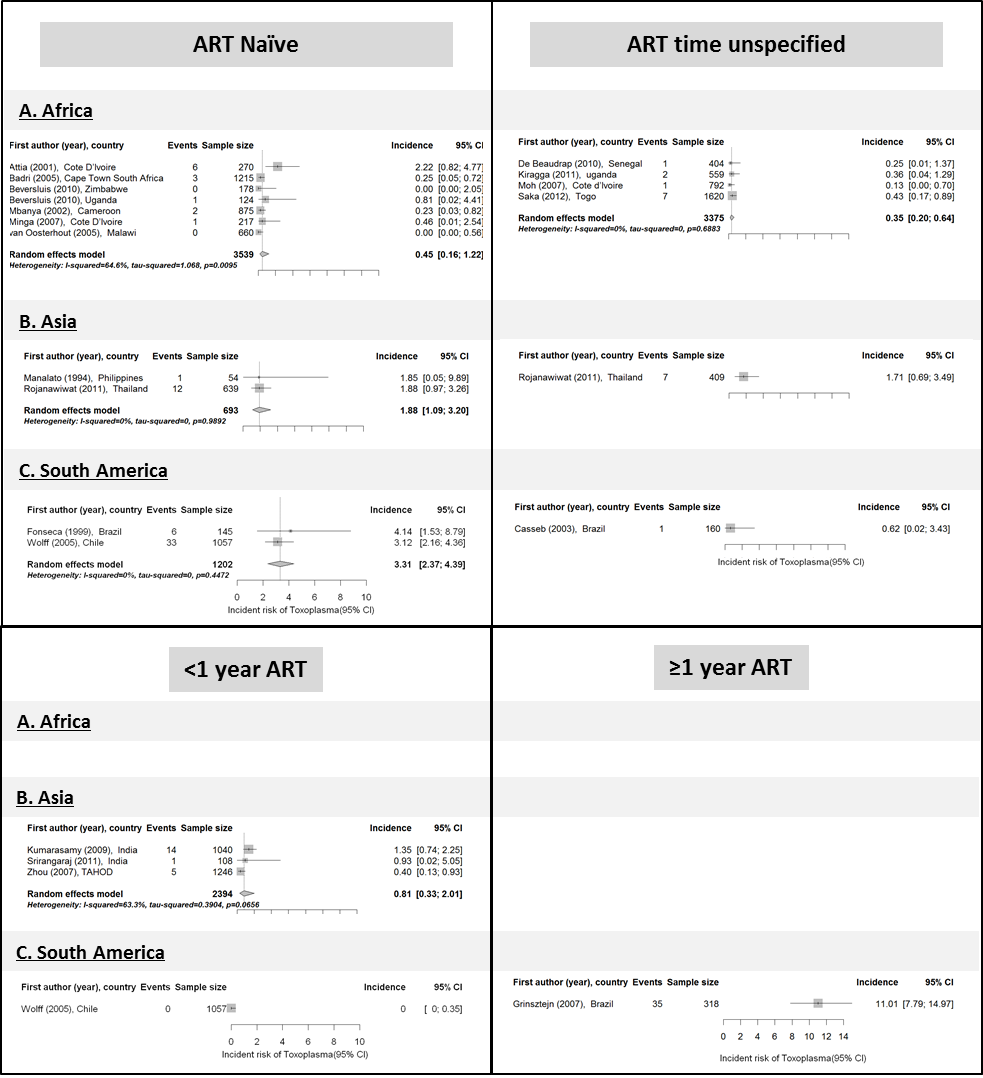
*

***Supplementary figure* 1i: Cryptosporidium diarrhoea**

*
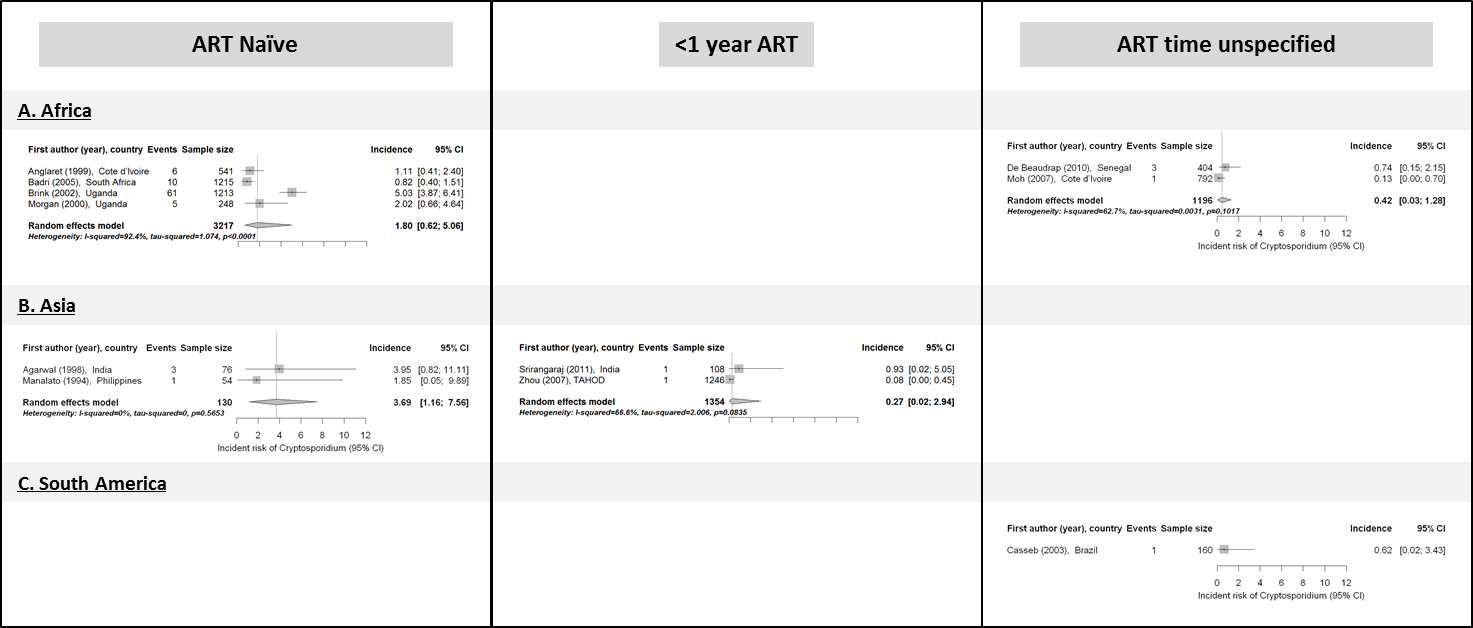
*

***Supplementary figure* 1j: Unspecified forms of tuberculosis**


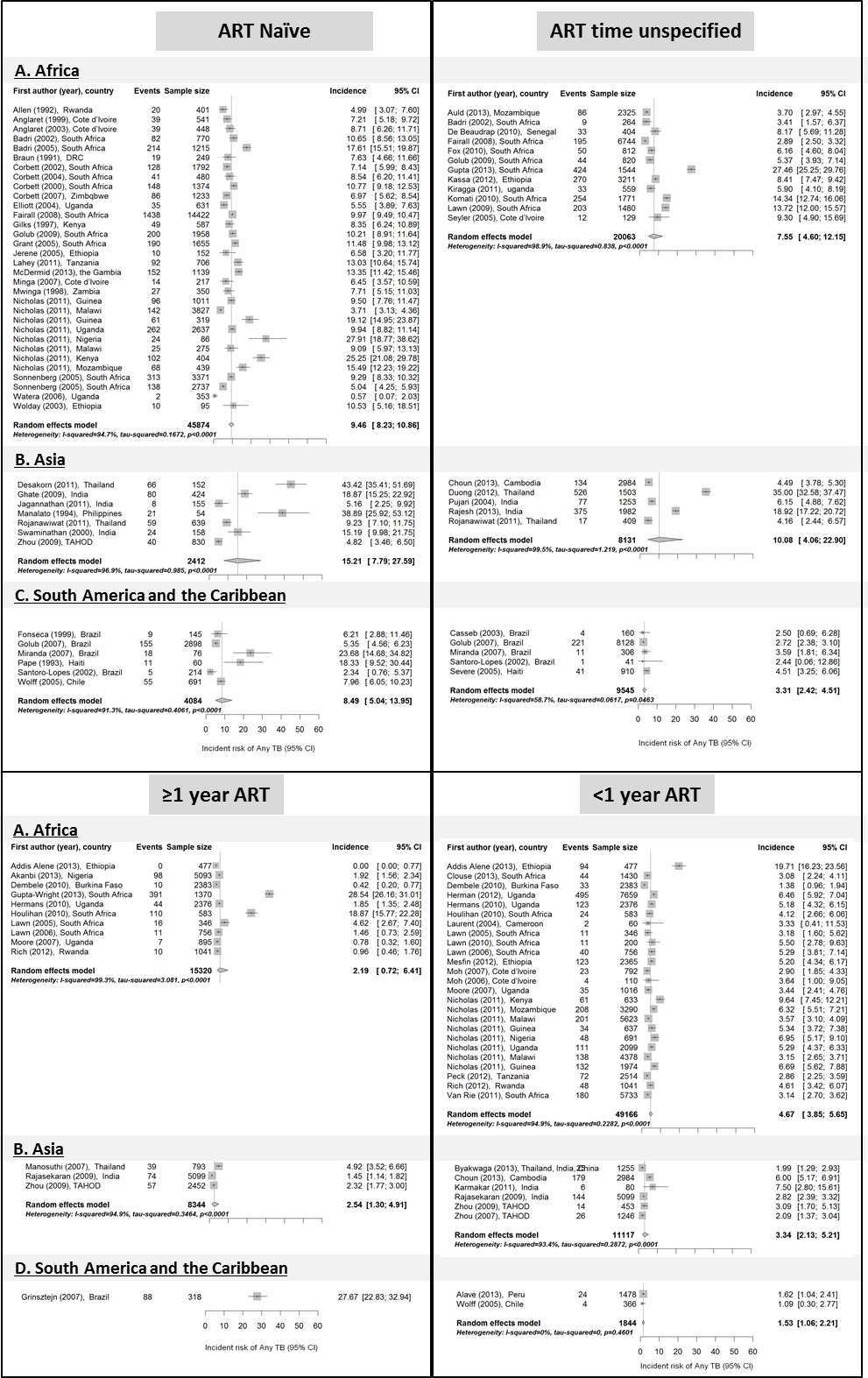


***Supplementary figure* 1k: Pulmonary tuberculosis**

***
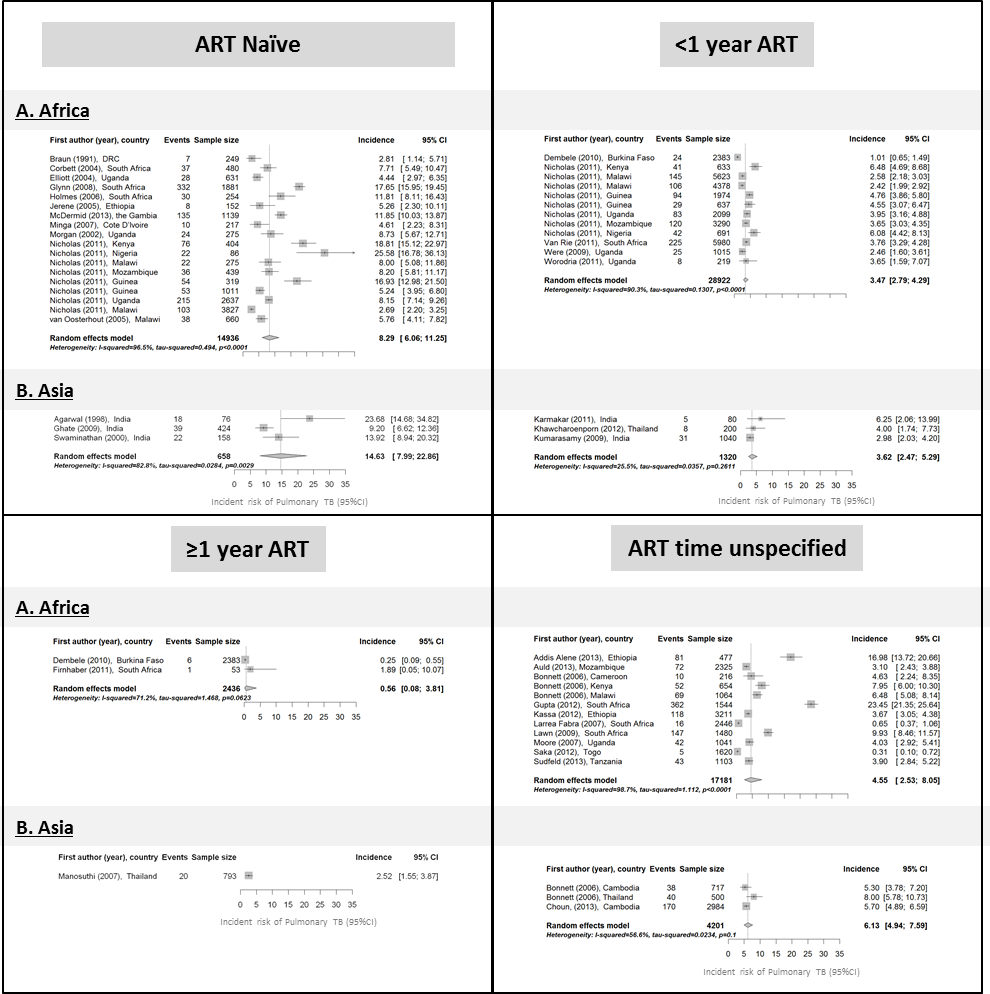
***

***Supplementary figure* 1l: Extra-Pulmonary tuberculosis**


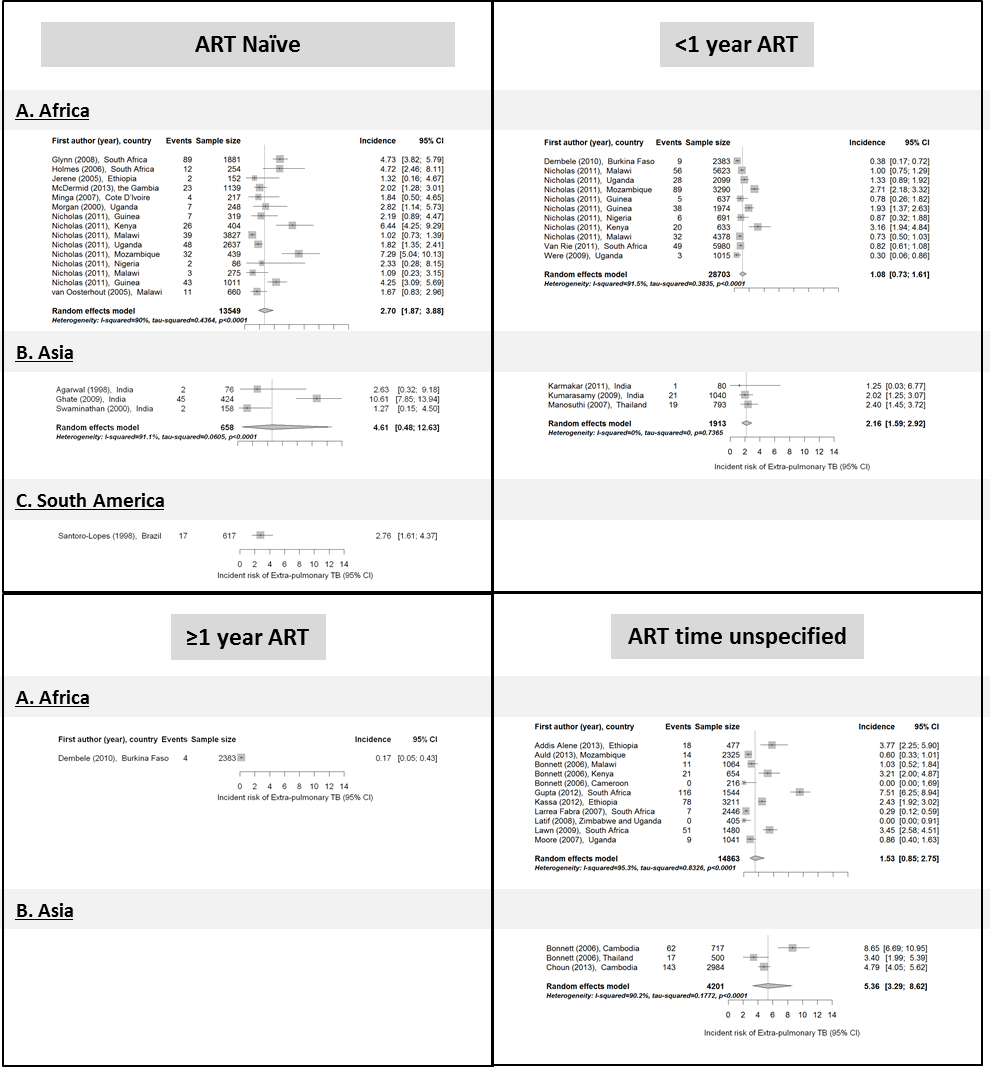


***Supplementary figure* 1m: Bacterial pneumonia**


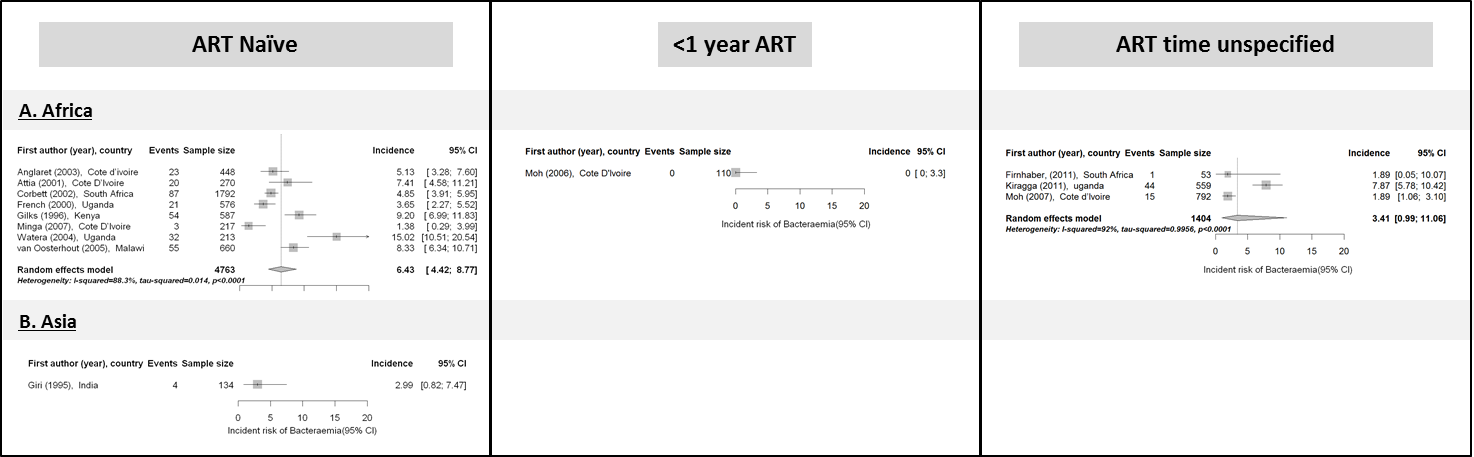


***Supplementary figure* 1n: Isolated bacteraemia**


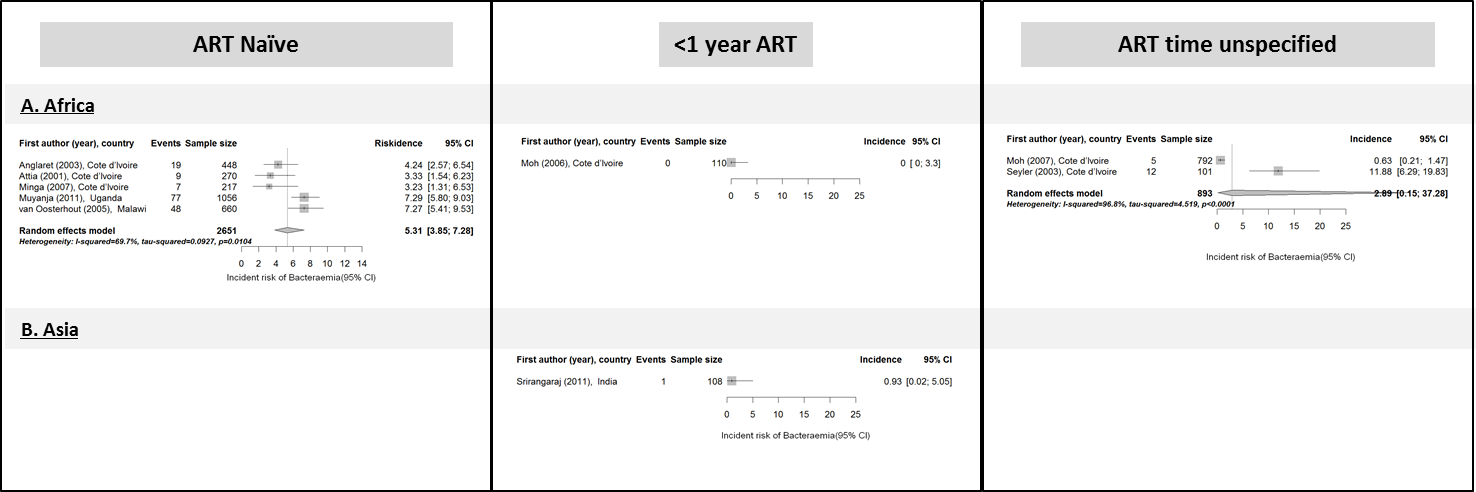


***Supplementary figure* 1o: Bacterial enteritis**


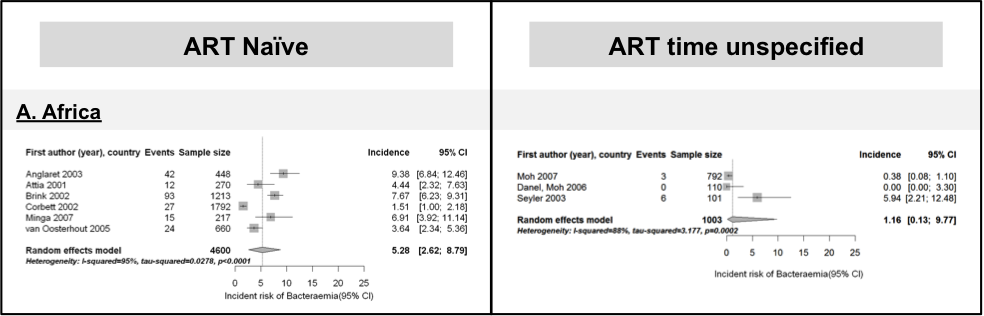


**Supplementary Table 1. Regional incidence of 11 opportunistic infections across ART categories in Sub-Saharan Africa, Asia and Latin America.**

|  | Number of studies | Summary Risk (95%CI) | I^2^ | p-het | Crude meta-regression^a^ | | Adjusted meta-regression^a,b^ | |
| --- | --- | --- | --- | --- | --- | --- | --- | --- |
|  |  |  |  |  | OR (95%CI) | P value |  | P value |
| Cryptococcus meningitis | | | | | | | | |
| Sub-Saharan Africa | 18 | 0.8% (0.4%-1.6%) | 96.6% | <0.001 | 1 | 0.30 | 1 | 0.03 |
| Asia | 11 | 1.6% (0.8%-3.3%) | 95.4% | <0.001 | 1.85 (0.82-4.20) |  | 2.71 (1.33-5.50) |  |
| Latin America & Caribbean | 5 | 1.8% (0.7%-4.4%) | 86.2% | <0.001 | 1.60 (0.52-5.00) |  | 1.60 (0.51-4.97) |  |
| PCP |  |  |  |  |  |  |  |  |
| Sub-Saharan Africa | 8 | 0.4% (0.2%-0.9%) | 93.1% | <0.001 | 1 | 0.01 | 1 | 0.002 |
| Asia | 10 | 2.1% (0.7%-5.8%) | 98.7% | <0.001 | 5.58 (1.58-19.67) |  | 9.88 (2.68-36.39) |  |
| Latin America & Caribbean | 7 | 4.1% (1.5%-10.7%) | 97.1% | <0.001 | 11.03 (2.89-42.11) |  | 10.57 (2.56-43.55) |  |
| Oral candidiasis |  |  |  |  |  |  |  |  |
| Sub-Saharan Africa | 12 | 6.8% (4.0%-11.2%) | 98.2% | <0.001 | 1 | 0.68 | 1 | 0.88 |
| Asia | 7 | 10.6% (4.0%-25.4%) | 98.3% | <0.001 | 1.66 (0.48-5.70) |  | 1.16 (0.52-2.59) |  |
| Latin America & Caribbean | 3 | 6.6% (0.5%-51.8%) | 99.1% | <0.001 | 1.03 (0.19-5.50) |  | 1.21 (0.33-4.41) |  |
| Oesophageal candidiasis |  |  |  |  |  |  |  |  |
| Sub-Saharan Africa | 9 | 1.4% (0.6%-3.0%) | 934% | <0.001 | 1 | 0.26 | 1 | 0.40 |
| Asia | 5 | 1.6% (0.5%-5.1%) | 87.0% | <0.001 | 1.32 (0.26-6.82) |  | 1.92 (0.29-12.82) |  |
| Latin America & Caribbean | 2 | 7.5% (2.1%-23.5%) | 89.2% | <0.001 | 5.92 (0.64-54.7) |  | 5.72 (0.25-129.3) |  |
| Herpes zoster/shingles |  |  |  |  |  |  |  |  |
| Sub-Saharan Africa | 8 | 7.7% (4.5%-12.8%) | 94.9% | <0.001 | 1 | 0.35 | 1 | 0.75 |
| Asia | 5 | 4.1% (1.8%-9.0%) | 94.5% | <0.001 | 0^.^52 (0.18-1.51) |  | 0.74 (0.26-2.13) |  |
| Latin America & Caribbean | 3 | 4.0% (1.0%-14.5%) | 96.9% | <0.001 | 0^.^53 (0.15-1.89) |  | 0.72 (0.20-2.55) |  |
| Herpes simplex/GUD |  |  |  |  |  |  |  |  |
| Sub-Saharan Africa | 13 | 5.1% (2.5%-10.2%) | 95.8% | <0.001 | 1 | 0.70 | 1 | 0.56 |
| Asia | 5 | 2.4% (0.9%-6.1%) | 96.5% | <0.001 | 0.49 (0.08-3.10) |  | 2.15 (0.46-10.02) |  |
| Latin America & Caribbean | 1 | 5.7% (3.4%-8.8%) | - | - | 1.18 (0.03-42.9) |  | 1.17 (0.13-10.25) |  |
| Kaposi’s sarcoma |  |  |  |  |  |  |  |  |
| Sub-Saharan Africa | 19 | 0.7% (0.5%-1.0%) | 92.9% | <0.001 | 1 | 0.26 | 1 | 0.25 |
| Asia | 1 | 1.3% (0.0%-7.1%) | - | - | 3.20 (0.10-106.82) |  | 1.98 (0.05-73.26) |  |
| Latin America & Caribbean | 5 | 2.0% (0.8%-4.9%) | 91.4% | <0.001 | 3.38 (0.69-16.49) |  | 4.51 (0.71-28.69) |  |
| Toxoplasmosis |  |  |  |  |  |  |  |  |
| Sub-Saharan Africa | 11 | 0.4% (0.2%-0.8%) | 52.5% | 0.02 | 1 | 0.03 | 1 | 0.01 |
| Asia | 6 | 1.3% (0.8%-2.0%) | 46.8% | 0.09 | 3.14 (0.95-10.33) |  | 7.46 (2.31-24.14) |  |
| Latin America & Caribbean | 5 | 2.6% (0.9%-7.0%) | 91.3% | <0.001 | 5.43 (1.52-19.44) |  | 2.69 (0.73-9.84) |  |
| Unspecified TB |  |  |  |  |  |  |  |  |
| Sub-Saharan Africa | 79 | 6.3% (5.4%-7.3%) | 98.1% | <0.001 | 1 | 0.68 | 1 | 0.43 |
| Asia | 21 | 7.1% (4.3%-11.6%) | 99.1% | <0.001 | 1.18 (0.73-1.91) |  | 1.18 (0.76-1.81) |  |
| Latin America & Caribbean | 14 | 5.3% (3.1%-8.8%) | 97.2% | <0.001 | 0.88 (0.49-1.58) |  | 0.78 (0.46-1.32) |  |
| Pulmonary TB |  |  |  |  |  |  |  |  |
| Sub-Saharan Africa | 44 | 5.1% (3.9%-6.6%) | 98.1% | <0.001 | 1 | 0.39 | 1 | 0.14 |
| Asia | 10 | 6.6% (4.6%-9.4%) | 90.6% | <0.001 | 1.34 (0.68-2.66) |  | 1.55 (0.86-2.80) |  |
| EPTB |  |  |  |  |  |  |  |  |
| Sub-Saharan Africa | 38 | 1.6% (1.2%-2.2%) | 94.4% | <0.001 | 1 | 0.06 | 1 | 0.02 |
| Asia | 9 | 3.9% (2.5%-6.0%) | 90.5% | <0.001 | 2.33 (1.15-4.75) |  | 2.35 (1.31-4.23) |  |
| Latin America & Caribbean | 1 | 2.8% (1.6%-4.4%) | - | - | 1.74 (0.27-11.27) |  | 1.56 (0.33-7.45) |  |

Meta-regression analysis was performed for OIs with 15 or greater studies.

^a^ calculate using random effect meta-regression.

b-adjusted for ART status and length of follow-up.

PCP, Pneumocystis jirovecii pneumonia; TB, tuberculosis.

**Appendix 4: References of included studies**

1. Anglaret X, Chene G, Attia A, Toure S, Lafont S, Combe P, et al. Early chemoprophylaxis with trimethoprim-sulphamethoxazole for HIV-1-infected adults in Abidjan, Cote d'Ivoire: a randomised trial. Cotrimo-CI Study Group. Lancet. 1999; **353**(9163): 1463-8.

2. Beversluis D, Musoke R, Mandima P, Nyamapfeni P, Chipato T, Mukasa J, et al. Incidence of Major Clinical Outcomes in a Cohort of Ugandan and Zimbabwean Women with Primary HIV-1 Infection. 17th Conference on Retroviruses and Opportunistic Infections; 2010.

3. Corbett EL, Churchyard GJ, Charalambos S, Samb B, Moloi V, Clayton TC, et al. Morbidity and mortality in South African gold miners: impact of untreated disease due to human immunodeficiency virus. Clin Infect Dis. 2002; **34**(9): 1251-8.

4. French N, Gray K, Watera C, Nakiyingi J, Lugada E, Moore M, et al. Cryptococcal infection in a cohort of HIV-1-infected Ugandan adults. AIDS. 2002; **16**(7): 1031-8.

5. Minga A, Danel C, Abo Y, Dohoun L, Bonard D, Coulibaly A, et al. Progression to WHO criteria for antiretroviral therapy in a 7-year cohort of adult HIV-1 seroconverters in Abidjan, Cote d'Ivoire. Bull World Health Organ. 2007; **85**(2): 116-23.

6. Morgan D, Malamba SS, Orem J, Mayanja B, Okongo M, Whitworth JA. Survival by AIDS defining condition in rural Uganda. Sex Transm Infect. 2000; **76**(3): 193-7.

7. van Oosterhout JJ, Laufer MK, Graham SM, Thumba F, Perez MA, Chimbiya N, et al. A community-based study of the incidence of trimethoprim-sulfamethoxazole-preventable infections in Malawian adults living with HIV. J Acquir Immune Defic Syndr. 2005; **39**(5): 626-31.

8. Jarvis JN, Lawn SD, Vogt M, Bangani N, Wood R, Harrison TS. Screening for cryptococcal antigenemia in patients accessing an antiretroviral treatment program in South Africa. Clin Infect Dis. 2009; **48**(7): 856-62.

9. Laurent C, Kouanfack C, Koulla-Shiro S, Nkoue N, Bourgeois A, Calmy A, et al. Effectiveness and safety of a generic fixed-dose combination of nevirapine, stavudine, and lamivudine in HIV-1-infected adults in Cameroon: open-label multicentre trial. Lancet. 2004; **364**(9428): 29-34.

10. De Beaudrap P, Etard JF, Diouf A, Ndiaye I, Ndeye GF, Sow PS, et al. Incidence and determinants of new AIDS-defining illnesses after HAART initiation in a Senegalese cohort. BMC Infect Dis. 2010; **10**: 179.

11. Badri M, Maartens G, Bekker LG, Wood R. The spectrum and prognosis of AIDS-defining illnesses in Cape Town. Southern African Journal of HIV Medicine. 2005; (19): 11-7.

12. Parkes-Ratanshi R, Wakeham K, Levin J, Namusoke D, Whitworth J, Coutinho A, et al. Primary prophylaxis of cryptococcal disease with fluconazole in HIV-positive Ugandan adults: a double-blind, randomised, placebo-controlled trial. Lancet Infect Dis. 2011; **11**(12): 933-41.

13. Fenner L, Reid SE, Fox MP, Garone D, Wellington M, Prozesky H, et al. Tuberculosis and the risk of opportunistic infections and cancers in HIV-infected patients starting ART in Southern Africa. Trop Med Int Health. 2013; **18**(2): 194-8.

14. Kiragga AN, Castelnuovo B, Schaefer P, Muwonge T, Easterbrook PJ. Quality of data collection in a large HIV observational clinic database in sub-Saharan Africa: implications for clinical research and audit of care. J Int AIDS Soc. 2011; **14**: 3.

15. Larrea Fabra R, Roque Acosta MdC. Efectividad de la terapia antirretroviral de alta eficacia en la atención del SIDA^ies. Rev cuba med. 2007; **46**(4).

16. Latif A, Hakim J, Robertson V, Reid A, Bulaya-Tembo R, Chimbetete C, et al. Fixed duration interruptions are inferior to continuous treatment in African adults starting therapy with CD4 cell counts < 200 cells/mul. AIDS. 2008; **22**(2): 237-47.

17. Espie E, Pinoges L, Balkan S, Chanchhaya N, Molfino L, Narom P, et al. Cryptococcal meningitis in HIV-infected patients: a longitudinal study in Cambodia. Trop Med Int Health. 2010; **15**(11): 1375-81.

18. Manosuthi W, Chaovavanich A, Tansuphaswadikul S, Prasithsirikul W, Inthong Y, Chottanapund S, et al. Incidence and risk factors of major opportunistic infections after initiation of antiretroviral therapy among advanced HIV-infected patients in a resource-limited setting. J Infect. 2007; **55**(5): 464-9.

19. Pujari SN, Patel AK, Naik E, Patel KK, Dravid A, Patel JK, et al. Effectiveness of generic fixed-dose combinations of highly active antiretroviral therapy for treatment of HIV infection in India. J Acquir Immune Defic Syndr. 2004; **37**(5): 1566-9.

20. Zhou J, Paton NI, Ditangco R. AIDS-defining illness diagnosed within 90 days after starting highly active antiretroviral therapy among patients from the TREAT Asia HIV Observational Database. Int J STD AIDS. 2007; **18**(7): 446-52.

21. Chariyalertsak S, Supparatpinyo K, Sirisanthana T, Nelson KE. A controlled trial of itraconazole as primary prophylaxis for systemic fungal infections in patients with advanced human immunodeficiency virus infection in Thailand. Clin Infect Dis. 2002; **34**(2): 277-84.

22. Ghate M, Deshpande S, Tripathy S, Nene M, Gedam P, Godbole S, et al. Incidence of common opportunistic infections in HIV-infected individuals in Pune, India: analysis by stages of immunosuppression represented by CD4 counts. Int J Infect Dis. 2009; **13**(1): e1-8.

23. Manaloto CR, Perrault JG, Caringal LT, Santiago EG, Wignall FS, Gonzales VL, et al. Natural history of HIV infection in Filipino female commercial sex workers. J Acquir Immune Defic Syndr. 1994; **7**(11): 1157-68.

24. Chim B, Piseth SS, Heng VC, Sopheak T, Lynen L, Griensven Jv. Integrated cryptococcal antigen screening and pre-emptive treatment prior to initiation of antiretroviral treatment in Cambodia. Journal of AIDS and Clinical Research. 2013; **4**(7).

25. Rojanawiwat A, Tsuchiya N, Pathipvanich P, Pumpradit W, Schmidt WP, Honda S, et al. Impact of the National Access to Antiretroviral Program on the incidence of opportunistic infections in Thailand. Int Health. 2011; **3**(2): 101-7.

26. Casseb J, Fonseca LA, Veiga AP, de Almeida A, Bueno A, Ferez AC, et al. AIDS incidence and mortality in a hospital-based cohort of HIV-1-seropositive patients receiving highly active antiretroviral therapy in Sao Paulo, Brazil. AIDS Patient Care STDS. 2003; **17**(9): 447-52.

27. Wolff MJ, Beltran CJ, Vasquez P, Ayala MX, Valenzuela M, Berrios G, et al. The Chilean AIDS cohort: a model for evaluating the impact of an expanded access program to antiretroviral therapy in a middle-income country--organization and preliminary results. J Acquir Immune Defic Syndr. 2005; **40**(5): 551-7.

28. Linares L, Paz J, Bustamante B. Cryptococcal antigenemia in HIV infected patients with a CD4 count <= 100 cell mm-3. Mycoses. 2012; **55**: 206-7.

29. Attia A, Huet C, Anglaret X, Toure S, Ouassa T, Gourvellec G, et al. HIV-1-related morbidity in adults, Abidjan, Cote d'Ivoire: a nidus for bacterial diseases. J Acquir Immune Defic Syndr. 2001; **28**(5): 478-86.

30. van Oosterhout JJ, Laufer MK, Perez MA, Graham SM, Chimbiya N, Thesing PC, et al. Pneumocystis pneumonia in HIV-positive adults, Malawi. Emerg Infect Dis. 2007; **13**(2): 325-8.

31. Saka B, Landoh DE, Kombate K, Mouhari-Toure A, Makawa MS, Patassi A, et al. [Evaluation of antiretroviral treatment in a cohort of 1,620 HIV-infected patients in Togo]. Medecine et sante tropicales. 2012; **22**(2): 193-7.

32. Giri TK, Pande I, Mishra NM, Kailash S, Uppal SS, Kumar A. Spectrum of clinical and laboratory characteristics of HIV infection in northern India. J Commun Dis. 1995; **27**(3): 131-41.

33. Byakwaga H, Petoumenos K, Ananworanich J, Zhang F, Boyd MA, Sirisanthana T, et al. Predictors of clinical progression in HIV-1-infected adults initiating combination antiretroviral therapy with advanced disease in the Asia-Pacific region: results from the TREAT Asia HIV observational database. Journal of the International Association of Providers of AIDS Care. 2013; **12**(4): 270-7.

34. Duong T, Jourdain G, Ngo-Giang-Huong N, Le Coeur S, Kantipong P, Buranabanjasatean S, et al. Laboratory and clinical predictors of disease progression following initiation of combination therapy in HIV-infected adults in Thailand. PLoS One. 2012; **7**(8): e43375.

35. Lim PL, Zhou J, Ditangco RA, Law MG, Sirisanthana T, Kumarasamy N, et al. Failure to prescribe pneumocystis prophylaxis is associated with increased mortality, even in the cART era: results from the Treat Asia HIV observational database. J Int AIDS Soc. 2012; **15**(1): 1.

36. Fonseca LA, Reingold AL, Casseb JR, Brigido LF, Duarte AJ. AIDS incidence and survival in a hospital-based cohort of asymptomatic HIV seropositive patients in Sao Paulo, Brazil. Int J Epidemiol. 1999; **28**(6): 1156-60.

37. Santoro-Lopes G, Harrison LH, Moulton LH, Lima LA, de Pinho AM, Hofer C, et al. Gender and survival after AIDS in Rio de Janeiro, Brazil. J Acquir Immune Defic Syndr Hum Retrovirol. 1998; **19**(4): 403-7.

38. Hermanides HS, Gras L, Winkel CN, Gerstenbluth I, Van Sighem A, De Wolf F, et al. The efficacy of combination antiretroviral therapy in hiv type 1-infected patients treated in curacao compared with antillean, surinam, and dutch HIV type 1-infected patients treated in the Netherlands. AIDS Research and Human Retroviruses. 2011; **27**(6): 605-12.

39. Grinsztejn B, Veloso VG, Pilotto JH, Campos DP, Keruly JC, Moore RD. Comparison of clinical response to initial highly active antiretroviral therapy in the patients in clinical care in the United States and Brazil. Journal of Acquired Immune Deficiency Syndromes. 2007; **45**(5): 515-20.

40. Morgan D, Mahe C, Mayanja B, Whitworth JA. Progression to symptomatic disease in people infected with HIV-1 in rural Uganda: prospective cohort study. BMJ. 2002; **324**(7331): 193-6.

41. Evans D, Maskew M, Sanne I. Increased risk of mortality and loss to follow-up among HIV-positive patients with oropharyngeal candidiasis and malnutrition before antiretroviral therapy initiation: a retrospective analysis from a large urban cohort in Johannesburg, South Africa. Oral surgery, oral medicine, oral pathology and oral radiology. 2012; **113**(3): 362-72.

42. Danel C, Moh R, Minga A, Anzian A, Ba-Gomis O, Kanga C, et al. CD4-guided structured antiretroviral treatment interruption strategy in HIV-infected adults in west Africa (Trivacan ANRS 1269 trial): a randomised trial. Lancet. 2006; **367**(9527): 1981-9.

43. Sudfeld CR, Giovannucci EL, Isanaka S, Aboud S, Mugusi FM, Wang M, et al. Vitamin D status and incidence of pulmonary tuberculosis, opportunistic infections, and wasting among HIV-infected Tanzanian adults initiating antiretroviral therapy. The Journal of infectious diseases. 2013; **207**(3): 378-85.

44. Desakorn V, Karmacharya BM, Thanachartwet V, Kyaw NL, Tansuphaswadikul S, Sahassananda D, et al. Effectiveness of fixed-dose combination stavudine, lamivudine and nevirapine (GPO-VIR) for treatment of naive HIV patients in Thailand: a 3-year follow-up. Southeast Asian J Trop Med Public Health. 2011; **42**(6): 1414-22.

45. Srirangaraj S, Venkatesha D. Opportunistic infections in relation to antiretroviral status among AIDS patients from south India. Indian J Med Microbiol. 2011; **29**(4): 395-400.

46. Rajesh R, Vidyasagar S, Varma DM, Naik A, Hegde BM, Guddattu V, et al. A prospective study of highly active antiretroviral therapy in Indian human immunodeficiency virus positive patients. The International journal of risk & safety in medicine. 2013; **25**(1): 53-65.

47. Seyler C, Anglaret X, Dakoury-Dogbo N, Messou E, Toure S, Danel C, et al. Medium-term survival, morbidity and immunovirological evolution in HIV-infected adults receiving antiretroviral therapy, Abidjan, Cote d'Ivoire. Antivir Ther. 2003; **8**(5): 385-93.

48. Iwuji CC, Mayanja BN, Weiss HA, Atuhumuza E, Hughes P, Maher D, et al. Morbidity in HIV-1-infected individuals before and after the introduction of antiretroviral therapy: a longitudinal study of a population-based cohort in Uganda. HIV medicine. 2011; **12**(9): 553-61.

49. Delany S, Mlaba N, Clayton T, Akpomiemie G, Capovilla A, Legoff J, et al. Impact of aciclovir on genital and plasma HIV-1 RNA in HSV-2/HIV-1 co-infected women: a randomized placebo-controlled trial in South Africa. AIDS. 2009; **23**(4): 461-9.

50. Mayaud P, Nagot N, Konate I, Ouedraogo A, Weiss HA, Foulongne V, et al. Effect of HIV-1 and antiretroviral therapy on herpes simplex virus type 2: a prospective study in African women. Sex Transm Infect. 2008; **84**(5): 332-7.

51. Moh R, Danel C, Messou E, Ouassa T, Gabillard D, Anzian A, et al. Incidence and determinants of mortality and morbidity following early antiretroviral therapy initiation in HIV-infected adults in West Africa. AIDS. 2007; **21**(18): 2483-91.

52. Reynolds SJ, Makumbi F, Newell K, Kiwanuka N, Ssebbowa P, Mondo G, et al. Effect of daily aciclovir on HIV disease progression in individuals in Rakai, Uganda, co-infected with HIV-1 and herpes simplex virus type 2: a randomised, double-blind placebo-controlled trial. Lancet Infect Dis. 2012; **12**(6): 441-8.

53. Kumarasamy N, Venkatesh K, Devaleenol B, Poongulali S, Yepthomi T, Saghayam S, et al. AIDS-defining illnesses after initiating generic HAART in South India: the case of earlier identification and intervention. 16th Conference on Retroviruses and Opportunistic Infections; 2009.

54. Mbulaiteye SM, Katabira ET, Wabinga H, Parkin DM, Virgo P, Ochai R, et al. Spectrum of cancers among HIV-infected persons in Africa: the Uganda AIDS-Cancer Registry Match Study. International journal of cancer Journal international du cancer. 2006; **118**(4): 985-90.

55. Mwachari CW, Shepherd BE, Cleopa O, Odhiambo JA, Cohen CR. Mortality and burden of disease in a cohort of HIV-seropositive adults in Nairobi, Kenya. Int J STD AIDS. 2004; **15**(2): 120-6.

56. Balkan S, O'Brien D, Humblet P, Olson D, Pujades-Rodriguez M. The burden of opportunistic infections in adults receiving anti-retroviral therapy in resource-limited settings in Medecins Sans Frontieres-supported projects. 14th Conference on Retroviruses and Opportunistic Infections; 2007.

57. Bohlius J, Valeri F, Maskew M, Prozesky H, Chimbetete C, Lumano-Mulenga P, et al. Incidence of Kaposi Sarcoma in HIV-infected patients-a prospective multi-cohort study from Southern Africa. Infectious Agents and Cancer. 2012; **7**.

58. Asiimwe F, Moore D, Were W, Nakityo R, Campbell J, Barasa A, et al. Clinical outcomes of HIV-infected patients with Kaposi's sarcoma receiving nonnucleoside reverse transcriptase inhibitor-based antiretroviral therapy in Uganda. HIV Med. 2012; **13**(3): 166-71.

59. Agarwal AK, Singh NY, Devi LB, Shyamkanhai KH, Singh YM, Bhattacharya SK. Clinical features & HIV progression as observed longitudinally in a cohort of injecting drug users in Manipur. Indian J Med Res. 1998; **108**: 51-7.

60. Mbanya DN, Zebaze R, Minkoulou EM, Binam F, Koulla S, Obounou A. Clinical and epidemiologic trends in HIV/AIDS patients in a hospital setting of Yaounde, Cameroon: a 6-year perspective. Int J Infect Dis. 2002; **6**(2): 134-8.

61. Brink AK, Mahe C, Watera C, Lugada E, Gilks C, Whitworth J, et al. Diarrhea, CD4 counts and enteric infections in a community-based cohort of HIV-infected adults in Uganda. J Infect. 2002; **45**(2): 99-106.

62. Anglaret X, Messou E, Ouassa T, Toure S, Dakoury-Dogbo N, Combe P, et al. Pattern of bacterial diseases in a cohort of HIV-1 infected adults receiving cotrimoxazole prophylaxis in Abidjan, Cote d'Ivoire. AIDS. 2003; **17**(4): 575-84.

63. Dembele M, Saleri N, Carvalho AC, Saouadogo T, Hien AD, Zabsonre I, et al. Incidence of tuberculosis after HAART initiation in a cohort of HIV-positive patients in Burkina Faso. Int J Tuberc Lung Dis. 2010; **14**(3): 318-23.

64. Seyler C, Toure S, Messou E, Bonard D, Gabillard D, Anglaret X. Risk factors for active tuberculosis after antiretroviral treatment initiation in Abidjan. Am J Respir Crit Care Med. 2005; **172**(1): 123-7.

65. Nicholas S, Sabapathy K, Ferreyra C, Varaine F, Pujades-Rodriguez M. Incidence of tuberculosis in HIV-infected patients before and after starting combined antiretroviral therapy in 8 sub-Saharan African HIV programs. J Acquir Immune Defic Syndr. 2011; **57**(4): 311-8.

66. McDermid JM, Hennig BJ, Sande Mvd, Hill AVS, Whittle HC, Jaye A, et al. Host iron redistribution as a risk factor for incident tuberculosis in HIV infection: an 11-year retrospective cohort study. BMC Infectious Diseases. 2013; **13**(48).

67. Akanbi MO, Achenbach CJ, Feinglass J, Taiwo B, Onu A, Pho MT, et al. Tuberculosis after one year of combination antiretroviral therapy in Nigeria: a retrospective cohort study. AIDS Res Hum Retroviruses. 2013; **29**(6): 931-7.

68. Gupta-Wright A, Wood R, Bekker LG, Lawn SD. Temporal association between incident tuberculosis and poor virological outcomes in a south african antiretroviral treatment service. Journal of Acquired Immune Deficiency Syndromes. 2013; **64**(3): 261-70.

69. Badri M, Wilson D, Wood R. Effect of highly active antiretroviral therapy on incidence of tuberculosis in South Africa: a cohort study. Lancet. 2002; **359**(9323): 2059-64.

70. Corbett EL, Charalambous S, Moloi VM, Fielding K, Grant AD, Dye C, et al. Human immunodeficiency virus and the prevalence of undiagnosed tuberculosis in African gold miners. Am J Respir Crit Care Med. 2004; **170**(6): 673-9.

71. Corbett EL, Bandason T, Cheung YB, Munyati S, Godfrey-Faussett P, Hayes R, et al. Epidemiology of tuberculosis in a high HIV prevalence population provided with enhanced diagnosis of symptomatic disease. PLoS Med. 2007; **4**(1): e22.

72. Corbett EL, Churchyard GJ, Clayton TC, Williams BG, Mulder D, Hayes RJ, et al. HIV infection and silicosis: the impact of two potent risk factors on the incidence of mycobacterial disease in South African miners. AIDS. 2000; **14**(17): 2759-68.

73. Fairall LR, Bachmann MO, Louwagie GM, van Vuuren C, Chikobvu P, Steyn D, et al. Effectiveness of antiretroviral treatment in a South African program: a cohort study. Arch Intern Med. 2008; **168**(1): 86-93.

74. Golub JE, Pronyk P, Mohapi L, Thsabangu N, Moshabela M, Struthers H, et al. Isoniazid preventive therapy, HAART and tuberculosis risk in HIV-infected adults in South Africa: a prospective cohort. AIDS. 2009; **23**(5): 631-6.

75. Grant AD, Charalambous S, Fielding KL, Day JH, Corbett EL, Chaisson RE, et al. Effect of routine isoniazid preventive therapy on tuberculosis incidence among HIV-infected men in South Africa: a novel randomized incremental recruitment study. JAMA. 2005; **293**(22): 2719-25.

76. Sonnenberg P, Glynn JR, Fielding K, Murray J, Godfrey-Faussett P, Shearer S. How soon after infection with HIV does the risk of tuberculosis start to increase? A retrospective cohort study in South African gold miners. J Infect Dis. 2005; **191**(2): 150-8.

77. Houlihan CF, Mutevedzi PC, Lessells RJ, Cooke GS, Tanser FC, Newell ML. The tuberculosis challenge in a rural South African HIV programme. BMC Infect Dis. 2010; **10**: 23.

78. Lawn SD, Badri M, Wood R. Tuberculosis among HIV-infected patients receiving HAART: long term incidence and risk factors in a South African cohort. AIDS. 2005; **19**(18): 2109-16.

79. Lawn SD, Myer L, Bekker LG, Wood R. Burden of tuberculosis in an antiretroviral treatment programme in sub-Saharan Africa: impact on treatment outcomes and implications for tuberculosis control. AIDS. 2006; **20**(12): 1605-12.

80. Lawn SD, Myer L, Edwards D, Bekker LG, Wood R. Short-term and long-term risk of tuberculosis associated with CD4 cell recovery during antiretroviral therapy in South Africa. AIDS. 2009; **23**(13): 1717-25.

81. Fox MP, Sanne IM, Conradie F, Zeinecker J, Orrell C, Ive P, et al. Initiating patients on antiretroviral therapy at CD4 cell counts above 200 cells/mu l is associated with improved treatment outcomes in South Africa. AIDS. 2010; **24**(13): 2041-50.

82. Gupta A, Wood R, Kaplan R, Bekker LG, Lawn SD. Tuberculosis incidence rates during 8 years of follow-up of an antiretroviral treatment cohort in South Africa: comparison with rates in the community. PLoS One. 2012; **7**(3): e34156.

83. Komati S, Shaw PA, Stubbs N, Mathibedi MJ, Malan L, Sangweni P, et al. Tuberculosis risk factors and mortality for HIV-infected persons receiving antiretroviral therapy in South Africa. AIDS. 2010; **24**(12): 1849-55.

84. Lawn SD, Kranzer K, Edwards DJ, McNally M, Bekker LG, Wood R. Tuberculosis during the first year of antiretroviral therapy in a South African cohort using an intensive pretreatment screening strategy. AIDS. 2010; **24**(9): 1323-8.

85. Van Rie A, Westreich D, Sanne I. Tuberculosis in patients receiving antiretroviral treatment: Incidence, risk factors, and prevention strategies. Journal of Acquired Immune Deficiency Syndromes. 2011; **56**(4): 349-55.

86. Clouse K, Pettifor A, Maskew M, Bassett J, Van Rie A, Gay C, et al. Initiating antiretroviral therapy when presenting with higher CD4 cell counts results in reduced loss to follow-up in a resource-limited setting. AIDS. 2013; **27**(4): 645-50.

87. Elliott AM, Hodsdon WS, Kyosiimire J, Quigley MA, Nakiyingi JS, Namujju PB, et al. Cytokine responses and progression to active tuberculosis in HIV-1-infected Ugandans: a prospective study. Trans R Soc Trop Med Hyg. 2004; **98**(11): 660-70.

88. Gilks CF, Godfrey-Faussett P, Batchelor BI, Ojoo JC, Ojoo SJ, Brindle RJ, et al. Recent transmission of tuberculosis in a cohort of HIV-1-infected female sex workers in Nairobi, Kenya. AIDS. 1997; **11**(7): 911-8.

89. Jerene D, Lindtjorn B. Disease progression among untreated HIV-infected patients in South Ethiopia: implications for patient care. MedGenMed. 2005; **7**(3): 66.

90. Watera C, Todd J, Muwonge R, Whitworth J, Nakiyingi-Miiro J, Brink A, et al. Feasibility and effectiveness of cotrimoxazole prophylaxis for HIV-1-infected adults attending an HIV/AIDS clinic in Uganda. J Acquir Immune Defic Syndr. 2006; **42**(3): 373-8.

91. Wolday D, Hailu B, Girma M, Hailu E, Sanders E, Fontanet AL. Low CD4+ T-cell count and high HIV viral load precede the development of tuberculosis disease in a cohort of HIV-positive ethiopians. Int J Tuberc Lung Dis. 2003; **7**(2): 110-6.

92. Hermans SM, Kiragga AN, Schaefer P, Kambugu A, Hoepelman AI, Manabe YC. Incident tuberculosis during antiretroviral therapy contributes to suboptimal immune reconstitution in a large urban HIV clinic in sub-Saharan Africa. PLoS One. 2010; **5**(5): e10527.

93. Moore D, Liechty C, Ekwaru P, Were W, Mwima G, Solberg P, et al. Prevalence, incidence and mortality associated with tuberculosis in HIV-infected patients initiating antiretroviral therapy in rural Uganda. AIDS. 2007; **21**(6): 713-9.

94. Allen S, Batungwanayo J, Kerlikowske K, Lifson AR, Wolf W, Granich R, et al. Two-year incidence of tuberculosis in cohorts of HIV-infected and uninfected urban Rwandan women. Am Rev Respir Dis. 1992; **146**(6): 1439-44.

95. Mwinga A, Hosp M, Godfrey-Faussett P, Quigley M, Mwaba P, Mugala BN, et al. Twice weekly tuberculosis preventive therapy in HIV infection in Zambia. AIDS. 1998; **12**(18): 2447-57.

96. Lahey T, Mitchell BK, Arbeit RD, Sheth S, Matee M, Horsburgh CR, et al. Polyantigenic interferon- gamma responses are associated with protection from TB among HIV-infected adults with childhood BCG immunization. PLoS ONE. 2011; **49**.

97. Auld AF, Mbofana F, Shiraishi RW, Alfredo C, Sanchez M, Ellerbrock TV, et al. Incidence and determinants of tuberculosis among adults initiating antiretroviral therapy--Mozambique, 2004-2008. PLoS One. 2013; **8**(1): e54665.

98. Kassa A, Teka A, Shewaamare A, Jerene D. Incidence of tuberculosis and early mortality in a large cohort of HIV infected patients receiving antiretroviral therapy in a tertiary hospital in Addis Ababa, Ethiopia. Trans R Soc Trop Med Hyg. 2012; **106**(6): 363-70.

99. Alene KA, Ansha N, Taye BW. Incidence and predictors of tuberculosis among adult people living with human immunodeficiency virus at the University of Gondar Referral Hospital, Northwest Ethiopia. BMC Infectious Diseases. 2013; **13**(292).

100. Rich ML, Miller AC, Niyigena P, Franke MF, Niyonzima JB, Socci A, et al. Excellent clinical outcomes and high retention in care among adults in a community-based HIV treatment program in rural Rwanda. Journal of acquired immune deficiency syndromes (1999). 2012; **59**(3): e35-42.

101. Hermans SM, van Leth F, Manabe YC, Hoepelman AI, Lange JM, Kambugu A. Earlier initiation of antiretroviral therapy, increased tuberculosis case finding and reduced mortality in a setting of improved HIV care: a retrospective cohort study. HIV Med. 2012; **13**(6): 337-44.

102. Mesfin N, Deribew A, Yami A, Solomon T, Van Geertruyden JP, Colebunders R. Predictors of antiretroviral treatment-associated tuberculosis in Ethiopia: a nested case-control study. Int J STD AIDS. 2012; **23**(2): 94-8.

103. Braun MM, Badi N, Ryder RW, Baende E, Mukadi Y, Nsuami M, et al. A retrospective cohort study of the risk of tuberculosis among women of childbearing age with HIV infection in Zaire. Am Rev Respir Dis. 1991; **143**(3): 501-4.

104. Swaminathan S, Ramachandran R, Baskaran G, Paramasivan CN, Ramanathan U, Venkatesan P, et al. Risk of development of tuberculosis in HIV-infected patients. Int J Tuberc Lung Dis. 2000; **4**(9): 839-44.

105. Zhou J, Elliott J, Li PC, Lim PL, Kiertiburanakul S, Kumarasamy N, et al. Risk and prognostic significance of tuberculosis in patients from The TREAT Asia HIV Observational Database. BMC Infect Dis. 2009; **9**: 46.

106. Rajasekaran S, Raja K, Jeyaseelan L, Vijilat S, Priya K, Mohan K, et al. Post-HAART tuberculosis in adults and adolescents with HIV in India: incidence, clinical and immunological profile. Indian J Tuberc. 2009; **56**(2): 69-76.

107. Jagannathan L, Chaturvedi M, Satish B, Satish KS, Desai A, Subbakrishna DK, et al. HLA-B57 and gender influence the occurrence of tuberculosis in HIV infected people of south India. Clinical & developmental immunology. 2011; **2011**: 549023.

108. Choun K, Thai S, Pe R, Lorent N, Lynen L, van Griensven J. Incidence and risk factors for tuberculosis in HIV-infected patients while on antiretroviral treatment in Cambodia. Trans R Soc Trop Med Hyg. 2013; **107**(4): 235-42.

109. Karmakar S, Sharma SK, Vashishtha R, Sharma A, Ranjan S, Gupta D, et al. Clinical characteristics of tuberculosis-associated immune reconstitution inflammatory syndrome in North Indian population of HIV/AIDS patients receiving HAART. Clinical & developmental immunology. 2011; **2011**: 239021.

110. Pape JW, Jean SS, Ho JL, Hafner A, Johnson WD, Jr. Effect of isoniazid prophylaxis on incidence of active tuberculosis and progression of HIV infection. Lancet. 1993; **342**(8866): 268-72.

111. Golub JE, Saraceni V, Cavalcante SC, Pacheco AG, Moulton LH, King BS, et al. The impact of antiretroviral therapy and isoniazid preventive therapy on tuberculosis incidence in HIV-infected patients in Rio de Janeiro, Brazil. AIDS. 2007; **21**(11): 1441-8.

112. Miranda A, Morgan M, Jamal L, Laserson K, Barreira D, Silva G, et al. Impact of antiretroviral therapy on the incidence of tuberculosis: the Brazilian experience, 1995-2001. PLoS One. 2007; **2**(9): e826.

113. Santoro-Lopes G, de Pinho AM, Harrison LH, Schechter M. Reduced risk of tuberculosis among Brazilian patients with advanced human immunodeficiency virus infection treated with highly active antiretroviral therapy. Clin Infect Dis. 2002; **34**(4): 543-6.

114. Severe P, Leger P, Charles M, Noel F, Bonhomme G, Bois G, et al. Antiretroviral therapy in a thousand patients with AIDS in Haiti. N Engl J Med. 2005; **353**(22): 2325-34.

115. Alave J, Paz J, González E, Campos M, Rodríguez M, Willig J, et al. Factores asociados a falla virológica en pacientes infectados con VIH que reciben terapia anti-retroviral en un hospital público del Perú^ies

Risk factors associated with virologic failure in HIV- infected patients receiving antiretroviral therapy at a public hospital in Peru^ien. Rev chil infectol. 2013; **30**(1): 42-8.

116. Brinkhof MW, Egger M, Boulle A, May M, Hosseinipour M, Sprinz E, et al. Tuberculosis after initiation of antiretroviral therapy in low-income and high-income countries. Clin Infect Dis. 2007; **45**(11): 1518-21.

117. Glynn JR, Murray J, Bester A, Nelson G, Shearer S, Sonnenberg P. Effects of duration of HIV infection and secondary tuberculosis transmission on tuberculosis incidence in the South African gold mines. AIDS. 2008; **22**(14): 1859-67.

118. Holmes CB, Wood R, Badri M, Zilber S, Wang B, Maartens G, et al. CD4 decline and incidence of opportunistic infections in Cape Town, South Africa: implications for prophylaxis and treatment. J Acquir Immune Defic Syndr. 2006; **42**(4): 464-9.

119. Were W, Moore D, Ekwaru P, Mwima G, Bunnell R, Kaharuza F, et al. A simple screening tool for active tuberculosis in HIV-infected adults receiving antiretroviral treatment in Uganda. Int J Tuberc Lung Dis. 2009; **13**(1): 47-53.

120. Worodria W, Massinga-Loembe M, Mayanja-Kizza H, Namaganda J, Kambugu A, Manabe YC, et al. Antiretroviral treatment-associated tuberculosis in a prospective cohort of HIV-infected patients starting ART. Clinical & developmental immunology. 2011; **2011**: 758350.

121. Firnhaber C, Azzoni L, Foulkes AS, Gross R, Yin X, van Amsterdam D, et al. Randomized trial of Time-Limited interruptions of protease Inhibitor-Based antiretroviral therapy (art) vs. continuous therapy for HIV-1 infection. PLoS ONE. 2011; **6**(6).

122. Bonnet MM, Pinoges LL, Varaine FF, Oberhauser BB, O'Brien DD, Kebede YY, et al. Tuberculosis after HAART initiation in HIV-positive patients from five countries with a high tuberculosis burden. AIDS. 2006; **20**(9): 1275-9.

123. Khawcharoenporn T, Apisarnthanarak A, Manosuthi W, Sungkanuparph S, Mundy LM. Isoniazid preventive therapy and 4-year incidence of pulmonary tuberculosis among HIV-infected Thai patients. Int J Tuberc Lung Dis. 2012; **16**(3): 336-41.

124. French N, Nakiyingi J, Carpenter LM, Lugada E, Watera C, Moi K, et al. 23-valent pneumococcal polysaccharide vaccine in HIV-1-infected Ugandan adults: double-blind, randomised and placebo controlled trial. Lancet. 2000; **355**(9221): 2106-11.

125. Gilks CF, Ojoo SA, Ojoo JC, Brindle RJ, Paul J, Batchelor BI, et al. Invasive pneumococcal disease in a cohort of predominantly HIV-1 infected female sex-workers in Nairobi, Kenya. Lancet. 1996; **347**(9003): 718-23.

126. Watera C, Nakiyingi J, Miiro G, Muwonge R, Whitworth JA, Gilks CF, et al. 23-Valent pneumococcal polysaccharide vaccine in HIV-infected Ugandan adults: 6-year follow-up of a clinical trial cohort. AIDS. 2004; **18**(8): 1210-3.

127. Muyanja SZ, Larke N, Rutebarika D, Kaddu I, Nakubulwa S, Levin J, et al. Decreasing trends of bacteraemia among HIV-infected Ugandan adults: incidence, aetiology, clinical outcomes and effect of antiretroviral therapy in a semi-urban setting (2000-2008). Trop Med Int Health. 2011; **16**(6): 756-65.
